# Bounds for spectral projectors on generic tori

**DOI:** 10.1007/s00208-022-02547-w

**Published:** 2022-12-23

**Authors:** Pierre Germain, Simon L. Rydin Myerson

**Affiliations:** 1 Courant Institute of Mathematical Sciences, New York University, 251 Mercer Street, New York, NY 10012-1185 USA; 2 Mathematics Institute, University of Warwick, Zeeman Building, Coventry, CV4 7AL United Kingdom

**Keywords:** 42A45, 11D75, 11L07, 42B15, 11H06, 11P21

## Abstract

We investigate norms of spectral projectors on thin spherical shells for the Laplacian on generic tori, including generic rectangular tori. We state a conjecture and partially prove it, improving on previous results concerning arbitrary tori.

## Introduction

### Boundedness of spectral projectors on Riemannian manifolds

Given a Riemannian manifold *M* with Laplace-Beltrami operator $\Delta$, and given some $\lambda \ge 1$ and $0<\delta <1$, let

1.1 $$\begin{aligned} P_{\lambda ,\delta } = P_{\lambda ,\delta }^{\chi } = \chi \left( \frac{\sqrt{-\Delta } - \lambda }{\delta } \right) , \end{aligned}$$

where $\chi$ is a cutoff function taking values in [0, 1] supported in $[-1,1]$, and equal to 1 on $[-\frac{1}{2},\frac{1}{2}]$. This definition is understood through the functional calculus for the Laplace-Beltrami operator, which is a self-adjoint operator on (complete) Riemannian manifolds.

A general question is to estimate

$$\begin{aligned} {\Vert P_{\lambda ,\delta }^\chi \Vert _{L^2 \rightarrow L^p}, \qquad \text {where}\ p \in [2,\infty ]}, \end{aligned}$$

the exact choice of $\chi$ being immaterial^1^.

The answer to this question is known in the case of the Euclidean space: define

$$\begin{aligned} p_{ST} = \frac{2(d+1)}{d-1}, \qquad \sigma (p) = d - 1 - \frac{2d}{p}. \end{aligned}$$

Then by Stein-Tomas [20, 21] we have

1.2 $$\begin{aligned} \Vert P_{\lambda ,\delta } \Vert _{L^2 \rightarrow L^p} \sim \left\{ \begin{array}{ll} \lambda ^{{\sigma (p)}/{2}} \delta ^{1/2} &{} \text {if}\ p \ge p_{ST}, \\ \lambda ^{\frac{d-1}{2} \left( \frac{1}{2} - \frac{1}{p} \right) } \delta ^{\frac{(d+1)}{2}\left( \frac{1}{2} - \frac{1}{p} \right) } &{} \text {if}\ 2 \le p \le p_{ST}, \end{array}\right. \end{aligned}$$

where we write $A \sim B$ if the two quantities *A* and *B* are such that $\frac{1}{C}A \le B \le CA$, for a constant *C* which depends only on the *d*. The answer is again known in the case of compact Riemannian manifolds when $\delta = 1$ (Sogge [19], Theorem 5.1.1), for which

1.3 $$\begin{aligned} \Vert P_{\lambda ,1} \Vert _{L^2 \rightarrow L^p} \sim \left\{ \begin{array}{ll} \lambda ^{{\sigma (p)}/2} &{} \text {if}\ p \ge p_{ST}, \\ \lambda ^{\frac{d-1}{2} \left( \frac{1}{2} - \frac{1}{p} \right) } &{} \text {if}\ 2 \le p \le p_{ST}. \end{array}\right. \end{aligned}$$

### Spectral projectors on tori

#### Different kinds of tori

From now on, we focus on the case of tori given by the quotient ${\mathbb {R}}^d / ({\mathbb {Z}} e_1 + \dots + {\mathbb {Z}} e_d)$, where $e_1,\dots ,e_d$ is a basis of ${\mathbb {R}}^d$, with the standard metric. This is equivalent to considering the operators

$$\begin{aligned} P_{\lambda ,\delta } = \chi \left( \frac{\sqrt{-{\mathcal {Q}}(\nabla )} - \lambda }{\delta } \right) \qquad \text {on} \;\; {\mathbb {T}}^d = {\mathbb {R}}^d / {\mathbb {Z}}^d, \end{aligned}$$

where $\nabla$ is the standard gradient operator, and ${\mathcal {Q}}$ is a quadratic form on ${\mathbb {R}}^d$, with coefficients $\beta _{ij}$:

$$\begin{aligned} {\mathcal {Q}}(x) = \sum _{i=1}^d \beta _{ij} x^i x^j \qquad \implies \qquad {{\mathcal {Q}}(\nabla ) = \sum _{i,j=1}^d \beta _{ij} \partial _i \partial _j}. \end{aligned}$$

Here $(\beta _{ij})$ is a symmetric positive definite real matrix. Dispensing with factors of $2\pi$, which can be absorbed in ${\mathcal {Q}}$, the associated Fourier multiplier has the symbol

$$\begin{aligned} \chi \left( \frac{\sqrt{{\mathcal {Q}}(k)} - \lambda }{\delta } \right) . \end{aligned}$$

Standard and rectangular tori correspond to the following particular cases.

- The *standard torus* corresponds to $(e_i)$ being orthonormal, or $\beta _{ij} = \delta _{ij}$.
- A *rectangular torus* corresponds to $(e_i)$ being orthogonal, or equivalently to a diagonal quadratic form $\beta _{ij} = \beta _i \delta _{ij}$.

We will be concerned in this article with generic tori, which for our purposes are defined as follows.

##### Definition 1.1

 

- Consider the rectangular tori with $\beta _i\in [1,2]$ for each *i*; we say a property is true for *generic rectangular tori* if it is true on a set of $(\beta _i)_{1\le i \le d}$ with full Lebesgue measure in $[1,2]^d$.
- Consider the tori with $\beta _{ij} = \delta _{ij} + h_{ij}$ for each $1\le i,j \le d$ and some $h_{ij}=h_{ji} \in [-\frac{1}{10d^2} , \frac{1}{10d^2} ]$; we say a property is true for *generic tori* if it is true for a set of $(h_{ij})_{1\le i\le j \le d}$ with full Lebesgue measure in $[-\frac{1}{10d^2} , \frac{1}{10d^2} ]^{d(d+1)/2}$.

#### The conjecture

It was conjectured in [10] that, for an arbitrary torus,

1.4 $$\begin{aligned} \Vert P_{\lambda ,\delta } \Vert _{L^{2} \rightarrow L^p} \lesssim 1 + (\lambda \delta )^{\frac{(d-1)}{2} \left( \frac{1}{2} - \frac{1}{p} \right) } + \lambda ^{\frac{d-1}{2} - \frac{d}{p}} \delta ^{1/2} \qquad \text {if} \ \delta > \lambda ^{-1}; \end{aligned}$$

here and below we denote $A \lesssim B$ if the quantities *A* and *B* are such that $A \le CB$ for a constant *C*, where *C* may depend on the dimension *d*. This paper also contains new results towards this conjecture, as well as a survey of known results

In the present paper, we turn our attention towards generic tori, for which the typical spacing between eigenvalues of $\sqrt{-\Delta }$ is $\lambda ^{1-d}$. Indeed, if *k* ranges in $[-R,R]^d$, then $\sqrt{{\mathcal {Q}}(k)}$ takes $(2R)^d$ values in $[-CR,CR]$; if the $\beta _{ij}$ are chosen generically we expect these to distribute approximately uniformly. This naturally leads to replacing the above conjecture by the following: for generic tori,

1.5 $$\begin{aligned} \Vert P_{\lambda ,\delta } \Vert _{L^{2} \rightarrow L^p} \lesssim _{\beta ,\epsilon } 1 + (\lambda \delta )^{\frac{(d-1)}{2} \left( \frac{1}{2} - \frac{1}{p} \right) } + \lambda ^{\frac{d-1}{2} - \frac{d}{p}} \delta ^{1/2} \qquad \text {if}\ \delta > \lambda ^{1-d+\epsilon } \end{aligned}$$

(here the notation $A \lesssim _\alpha B$ means that the constant *C* in the relation $A \le C B$ may depend on the parameter $\alpha$).

#### Known results if $p=\infty$

For $p=\infty$, the problem of bounding $\Vert P_{\lambda ,\delta }\Vert _{L^1 \rightarrow L^\infty }$ is closely related to counting lattice points in ellipsoids, a classical question in number theory. Namely, choosing $\chi = \textbf{1}_{[-1,1]}$,

$$\begin{aligned} \Vert P_{\lambda ,\delta } \Vert _{L^1 \rightarrow L^\infty } = N(\lambda + \delta ) - N(\lambda - \delta ), \end{aligned}$$

where $N(\lambda )$ is the counting function associated to the quadratic form ${\mathcal {Q}}$, defined as the number of lattice points $n \in {\mathbb {Z}}^d$ such that ${\mathcal {Q}}(n) < \lambda ^2$.

To leading order, $N(\lambda )$ equals $\textrm{Vol}(E) \lambda ^d$, where $\textrm{Vol}(E)$ is the ellipsoid $\{{\mathcal {Q}}(x) < 1\}$; the error term is denoted $P(\lambda )$:

$$\begin{aligned} N(\lambda ) = \textrm{Vol}(B_1) \lambda ^d + P(\lambda ). \end{aligned}$$

For the state of the art regarding $P(\lambda )$ for any fixed ${\mathcal {Q}}$ we refer the reader to the comments after (1.3) in [10], and to the work of Bourgain-Watt [7] giving an improved bound for the standard two-dimensional torus. For generic quadratic forms, there are a number of additional results.

- For generic diagonal forms, Jarník [13] showed that $P(\lambda ) = O (\lambda ^{d/2})$ if $d \ge 4$; a weaker, but more general, result is due to Schmidt [17].
- Landau [16] showed that $P(\lambda ) = \Omega (\lambda ^{\frac{d-1}{2}})$ for generic forms.
- It has been shown that the average size of the error, say $[{\mathbb {E}} |P(\lambda )|^2]^{1/2}$ is $O(\lambda ^{\frac{d-1}{2}})$, for different types of averaging: over translations of the integer lattice [15], over shears [14], and over the coefficients $(\beta _i)$ of a diagonal form [12].
- When $d=2$, Trevisan [22] has investigated in more detail the distribution of the normalised error $P(\lambda )\lambda ^{-1/2}$ when ${\mathcal {Q}}$ is chosen at random and $\lambda$ is large.
- The quantity $P(\lambda +\delta )-P(\lambda -\delta )$ has also received attention. In particular it has average size $O(\sqrt{\delta \lambda ^{d-1}})$ when averaged over translations of the integer lattice [9], provided that $\delta \le \lambda ^{-\frac{d-1}{d+1}-\epsilon }$.

These results lead to the conjecture that the correct bound for the error term $P(\lambda )$ for generic ${\mathcal {Q}}$, or for generic diagonal ${\mathcal {Q}}$, would be $O(\lambda ^{\frac{d-1}{2}})$. Meanwhile for $P(\lambda +\delta )-P(\lambda -\delta )$ the corresponding conjecture would be $O(\sqrt{\delta \lambda ^{d-1}})$, at least for $\delta >\lambda ^{1-d+\epsilon }$.

#### Known results if $p<\infty$

After the pioneering work of Zygmund [23], Bourgain [1] asked for $L^p$ bounds for eigenfunctions of the Laplacian on the (standard) torus. He conjectured that, if $\varphi$ is an eigenfunction of the Laplacian with eigenvalue $\lambda$, then

1.6 $$\begin{aligned} \Vert \varphi \Vert _{L^p} \lesssim \lambda ^{\frac{d}{2} - 1 - \frac{d}{p}} \Vert \varphi \Vert _{L^2} \qquad \text {if}\; \;p \ge \frac{2d}{d-2}, \end{aligned}$$

which is equivalent to the case $\delta = \lambda ^{-1}$ of (1.4) for the standard torus. Progress on this conjecture appeared in a series of articles [2–4] culminating in the proof of the $\ell ^2$-decoupling conjecture by Bourgain and Demeter [5], which implies (1.6) if $p \ge \frac{2(d-1)}{d-3}$ and $d \ge 4$.

Bounds for spectral projectors are essentially equivalent to bounds for the resolvent $(-\Delta + z)^{-1}$. This was the point of view adopted in Shen [18], Bourgain-Shao-Sogge-Yau [6], and Hickman [11]. Here the goal is to prove a sharp bound when $p^*=\frac{2d}{d-2}$ and $\delta$ is sufficiently large.

Finally, the authors of the present paper were able to prove the conjecture (1.4) when $\delta$ is sufficently large by combining $\ell ^2$-decoupling with a geometry of numbers argument [10].

To the best of our knowledge, all works concerned with $p<\infty$ address either the case of standard tori, or the general case of arbitrary tori; the generic case does not seem to have been considered specifically. This will be a focus of the present paper.

### A new result through harmonic analysis

The conjecture (1.5) was proved in [10] for arbitrary tori and $\delta$ not too small, and for generic tori we can improve the range for $\delta$ as follows.

#### Theorem 1.2

For generic rectangular tori and for generic tori (in the sense of Definition 1.1), the conjecture (1.5) is verified if $p>p_{ST}$ and, for some $\epsilon >0$,

$$\begin{aligned} \text {either}\ \delta > \max \left( \lambda ^{-1}, \lambda ^{\frac{p}{p-2} \left[ 1 - \frac{d}{2} + \frac{1}{p} \left( \frac{d^2 - d -2}{d-1} \right) \right] + \epsilon } \right) \quad \text {or}\ \lambda ^{1 - \frac{d}{2} + \frac{1}{p} \frac{d(d-3)}{d-1}+\epsilon }< \delta < \lambda ^{-1}. \end{aligned}$$

Namely, under these conditions, there holds for almost all choices of $(\beta _i)_{1\le i \le d}$ (for generic rectangular tori) or $(\beta _{ij})_{1\le i,j \le d}$ (for generic tori)

$$\begin{aligned} \Vert P_{\lambda ,\delta } \Vert _{L^2 \rightarrow L^p} \lesssim _{\beta ,\epsilon } \lambda ^{\frac{d-1}{2} - \frac{d}{p}} \delta ^{1/2}. \end{aligned}$$

In the particularly well-behaved case when $p=\infty$ and we consider generic diagonal tori, the theorem matches the classical result of Jarník [13] mentioned in the first bullet point in Sect. 1.2.3, which even promotes the upper bound in the theorem to an asymptotic in that case.

The proof of this theorem will be given in Sect. 4. The idea of this proof is to first express the spectral projector through the Schrödinger group. First note that the operator $\chi \left( \frac{ \sqrt{-{\mathcal {Q}}(\nabla )} - \lambda }{\delta }\right)$ can also be written $\chi \left( \frac{ -{\mathcal {Q}}(\nabla ) - \lambda ^2}{\lambda \delta } \right)$ by adapting the compactly supported function $\chi$. This in turn can be expressed as

$$\begin{aligned} P_{\lambda ,\delta } = \lambda \delta \int _{{\mathbb {R}}} \widehat{\chi }(\lambda \delta t) e^{-2 \pi i\lambda ^2 t} e^{-2\pi i t {\mathcal {Q}}(\nabla )} \,dt, \end{aligned}$$

and then split off into two pieces, corresponding to $|t| \lesssim \lambda ^{-1}$ and $|t| \gtrsim \lambda ^{-1}$ respectively:

$$\begin{aligned} P_{\lambda ,\delta }&= \int \lambda \delta \widehat{\chi }(\lambda t) \widehat{\chi }(\lambda \delta t) e^{-2\pi i\lambda ^2 t} e^{-2\pi i t {\mathcal {Q}}(\nabla )} \,dt \\&\quad + \int \lambda \delta [1-\widehat{\chi }(\lambda t)] \widehat{\chi }(\lambda \delta t) e^{-2\pi i\lambda ^2 t} e^{-2\pi i t {\mathcal {Q}}(\nabla )} \,dt \\&= P_{\lambda ,\delta }^{\textrm{small}} + P_{\lambda ,\delta }^{\textrm{large}}. \end{aligned}$$

It is easy to see that the operator $P_{\lambda ,\delta }^{\textrm{small}}$ can be written in the form $\delta P_{\lambda ,1}$ (after adjusting the cutoff function); in other words, this corresponds to the case $\lambda =1$, to which the universal bounds of Sogge apply.

Turning to the term $P_{\lambda ,\delta }^{\textrm{large}}$, its operator norm will be obtained through interpolation between

- A bound $L^{p_{ST}'} \rightarrow L^{p_{ST}}$, Theorem 4.1 below. As noted in [10], is a direct consequence of $\ell ^2$ decoupling (and valid for any torus).
- A bound $L^1 \rightarrow L^\infty$, for which genericity will be used. Namely, we will prove in Sect. 3 that, generically in $(\beta _{ij})$,
  1.7 $$\begin{aligned} \int _{1/N}^T \Vert \chi ( N^{-2} \Delta ) e^{it\Delta } \Vert _{L^1 \rightarrow L^\infty } \,dt \lesssim _{\beta ,\epsilon } T N^{\frac{d}{2}+\epsilon }. \end{aligned}$$

One could think of (1.7) as square-root cancellation in $L^1 L^\infty$ since $\Vert \chi ( N^{-2} \Delta ) \Vert _{L^1 \rightarrow L^\infty } \sim N^d$. One could also see this as a minor-arc type bound in the spirit of the circle method; indeed in the case $p=\infty$ the proof in effect reduces to an application of the Davenport-Heilbronn circle method.

### An elementary approach for $p=\infty$ and $\delta$ very small

When $\delta$ is small enough a more elementary counting argument can be used.

Our main result there is Theorem 6.1 below. We first state three $L^1\rightarrow L^\infty$ bounds proved at the start of Sect. 6 since they are particularly simple; we will then mention consequences for $L^1\rightarrow L^p$ bounds.

#### Theorem 1.3

For generic tori, and also for generic rectangular tori, the following holds. If $\delta < \lambda ^{1-2d-\epsilon }$, then

1.8 $$\begin{aligned} \Vert P_{\lambda ,\delta } \Vert _{L^1\rightarrow L^\infty } \lesssim _{\beta ,\epsilon } 1. \end{aligned}$$

If instead $a\in {\mathbb {Z}}$ with $d\le a\le 2d$ and $\lambda ^{-a}\le \delta \le \lambda ^{1-a}$, then

1.9 $$\begin{aligned} \Vert P_{\lambda ,\delta } \Vert _{L^1\rightarrow L^\infty } \lesssim _{\beta ,\epsilon } {\delta ^{1-\frac{a+1}{d+a+1}} \lambda ^{ d -1+\frac{a+1}{d+a+1}+ \epsilon }}. \end{aligned}$$

Finally if $a\in {\mathbb {Z}}$ with $\frac{d-1}{2}\le a<d$ and $\lambda ^{-a}\le \delta \le \lambda ^{1-a}$, we have

1.10 $$\begin{aligned} \Vert P_{\lambda ,\delta } \Vert _{L^1\rightarrow L^\infty } \lesssim _{\beta ,\epsilon } \delta ^{1- \frac{1}{d+1-a}} \lambda ^{d - 1 + {\frac{1}{d+1-a}} + \epsilon }. \end{aligned}$$

We remark that for $\delta \le \lambda ^{-1}$, interpolation with the $L^{p_{ST}'}\rightarrow L^{p_{ST}}$ bound from [10] gives

$$\begin{aligned} \Vert P_{\lambda ,\delta } \Vert _{L^2\rightarrow L^p} \lesssim _{\beta ,\epsilon } \lambda ^\epsilon ( \Vert P_{\lambda ,\delta } \Vert _{L^1\rightarrow L_\infty })^{\frac{1}{2}-\frac{p_{ST}}{2p}}. \end{aligned}$$

This would always fall short of the conjecture (1.5) for $p<\infty$, even with an optimal $L^1\rightarrow L_\infty$ bound. We highlight a few features of these bounds.

- In the setting of Theorem 1.3, conjecture (1.5) would give $\Vert P_{\lambda ,\delta } \Vert _{L^1\rightarrow L^\infty }\lesssim _{\beta ,\epsilon } 1+\delta \lambda ^{d-1+\epsilon }$.
- Although (1.9) and (1.10) do not recover (1.5), they do improve on the best known bounds for $N(\lambda -\delta )-N(\lambda +\delta )$ coming from the results listed in Sect. 1.2.3.
- Both (1.10) and (1.9) are special cases of the stronger estimate (6.1) below, while (1.8) has a short self-contained proof.
- We restrict to $a>\frac{d}{2}-1$ in (1.10) and to $a\le 2d$ in (1.10) solely becuase the remaining range is already covered by Theorem 1.2 or by (1.8), see also (6.2) below.
- When $a=d$ the bound (1.10) would be trivial, and hence for $a\ge d$ the bound (1.9) takes over.

In the proof of Theorem 6.1 we will use the Borel-Cantelli lemma to reduce to estimates for moments of $\Vert P_{\lambda ,\delta }\Vert$, where the moments are taken over $\lambda$ and $\beta$. A short computation reduces this to the following problem.

#### Problem 1.4

Estimate (from above, or asymptotically) the number of matrices of the form

$$\begin{aligned} P=\left( \begin{matrix} m^2_{11}&{}\cdots &{}m^2_{1b}\\ \vdots &{}\ddots &{}\vdots \\ m^2_{d1}&{}\cdots &{}m^2_{db}\\ \lambda ^2&{}\cdots &{}\lambda ^2 \end{matrix}\right) , \end{aligned}$$

where the $m_{ij}$ are integers, all entries in each row lie in a specified dyadic range, and also for each *k* the maximal $k\times k$ subdeterminant of *P* lies in a specified dyadic range.

In Sect. 6.2 we give an upper bound in this counting problem using what is in effect linear algebra, relying on the rather technical Lemma 5.7 below. We are then left with a maximum over all possible choices of the various dyadic ranges, and estimating this maximum will be the most challenging part of the proof.

The bound from Lemma 5.7 could be improved. See Remark 6.2 for one path. Another route concerns the case when all $\beta _{ij}$ are generic, that is the case of generic tori as opposed to generic rectangular tori. Then one could expand *P*; in place of the squares $m_{1i}^2,\dotsc ,m_{di}^2$ the *i*th column would contain all degree 2 monomials in $m_{1i},\dotsc ,m_{di}$. This should allow a smaller bound.

## Notations

We adopt the following normalizations for the Fourier series on ${\mathbb {T}}^d$ and Fourier transform on ${\mathbb {R}}^d$, respectively:

$$\begin{aligned}&f(x) = \sum _{k \in {\mathbb {Z}}^d} \widehat{f}_k e^{2\pi i k \cdot x}, \qquad \qquad \widehat{f}_k = \int _{{\mathbb {T}}^d} f(x) e^{-2\pi i k \cdot x} \,dx, \\&\widehat{f}(\xi ) = \int _{{\mathbb {R}}^d} f(x) e^{-2\pi ix \cdot \xi } \,dx, \qquad \qquad f(x) = \int _{{\mathbb {R}}^d} \widehat{f}(\xi ) e^{2\pi ix \cdot \xi } \,dx. \end{aligned}$$

With this normalization,

$$\begin{aligned} {\widehat{fg} = \widehat{f} * \widehat{g} \qquad \text {and} \qquad \widehat{f * g} = \widehat{f} \widehat{g},} \end{aligned}$$

and the Parseval and Plancherel theorems, respectively, are given by

$$\begin{aligned} {\Vert f \Vert _{L^2({\mathbb {T}}^d)} = \Vert \widehat{f} \Vert _{\ell ^2 ({\mathbb {Z}}^d)} \qquad \text {and} \qquad \Vert f \Vert _{L^2({\mathbb {R}}^d)} = \Vert \widehat{f} \Vert _{L^2 ({\mathbb {R}}^d)}.} \end{aligned}$$

The operator $m(\sqrt{-{\mathcal {Q}}(\nabla )})$ can be expressed as a Fourier multiplier

$$\begin{aligned} m(\sqrt{-{\mathcal {Q}}(\nabla )}) f = \sum _k m(\sqrt{{\mathcal {Q}}(k)}) \widehat{f}_k e^{2\pi i k \cdot x}, \end{aligned}$$

or through a convolution kernel

$$\begin{aligned} m(\sqrt{-{\mathcal {Q}}(\nabla )}) f (x) = \int K(x-y) f(y) \,dy, \quad \text {with} \quad K(z) = \sum _k m(\sqrt{{\mathcal {Q}}(k)}) e^{2\pi i k \cdot z}. \end{aligned}$$

In Sects. 5 and 6 we will often join together several matrices $A_1,\dotsc ,A_n$ with the same number of rows to make a larger matrix, for which we use the notation $(A_1\mid \cdots \mid A_n)$. We view column vectors as matrices with one column, so that $(A\mid \vec {v})$ is the matrix *A* with the vector $\vec {v}$ added as an extra column on the right.

Also in Sects. 5 and 6 we use the following notation relating to subdeterminants. If $k \le \min (p,q)$ and *M* is a matrix in ${\mathbb {R}}^{p\times q}$, we will denote $D_k(M)$ the maximum absolute value of a $k\times k$ subdeterminant of *M*:

$$\begin{aligned} D_k(M) = \max _{\begin{array}{c} {\mathcal {I}}\subset \{1,\dotsc , p\} \\ {\mathcal {J}}\subset \{1,\dotsc ,q\} \\ \#{\mathcal {I}}=\#{\mathcal {J}}=k \end{array}} \big |\det \, (M_{ij})_{i\in {\mathcal {I}},j\in {\mathcal {J}}}\big |. \end{aligned}$$

We further define $D_0(M)=1$ for ease of notation, and we let $D^{(\ell )}_k(M)$ denote the maximal subdeterminant, when the matrix *M* is restricted to its first $\ell$ columns:

$$\begin{aligned} D^{(\ell )}_k(M) =\max _{\begin{array}{c} {\mathcal {I}}\subset \{1,\dotsc , p\} \\ {\mathcal {J}}\subset \{1,\dotsc ,\ell \} \\ \#{\mathcal {I}}=\#{\mathcal {J}}=k \end{array}} \big |\det \, (M_{ij})_{i\in {\mathcal {I}},j\in {\mathcal {J}}}\big |. \end{aligned}$$

Given two quantities *A* and *B*, we denote $A \lesssim B$ if there exists a constant *C* such that $A \le CB$, and $A \sim B$ if $A \lesssim B$ and $B \lesssim A$. If the implicit constant *C* is allowed to depend on *a*, *b*, *c*, the notation becomes $A \lesssim _{a,b,c} B$. In the following, it will often be the case that the implicit constant will depend on $\beta$, and on an arbitrarily small power of $\lambda$, for instance $A \lesssim _{\beta ,\epsilon } \lambda ^\epsilon B$. When this is clear from the context, we simply write $A \lesssim \lambda ^\epsilon B$. Implicit constants in this notation may always depend on the dimension *d* of the torus that is the object of our study.

Finally, the Lebesgue measure of a set *E* is denoted $\textrm{mes}E$.

## Bounds on Weyl sums

Consider the smoothly truncated Weyl sum (or regularized fundamental solution for the anisotropic Schrödinger equation)

$$\begin{aligned} K_N(t,x) = \sum _{k\in {\mathbb {Z}}^d}e^{- 2\pi i(x\cdot k+t {\mathcal {Q}}(k))}\phi \bigg (\frac{k_1}{N}\bigg ) \dots \phi \bigg (\frac{k_d}{N}\bigg ), \end{aligned}$$

where $\phi$ is a smooth cutoff function supported on $[-1,1]$, equal to 1 on $[-\frac{1}{2},\frac{1}{2}]$.

In dimension one, it becomes

$$\begin{aligned} K^{(1)}_N(t,y) = \sum _{k\in {\mathbb {Z}}}e^{2\pi i(yk+k^2t)}\phi \bigg (\frac{k}{N}\bigg ). \end{aligned}$$

### Bound for small time

For small *t*, the following bound holds on any torus.

#### Lemma 3.1

For any non-degenerate quadratic form ${\mathcal {Q}}$, if $|t| \lesssim \frac{1}{N}$,

$$\begin{aligned} |K_N(t,x)| \lesssim \left( \frac{N}{tN + \frac{1}{N}} \right) ^{d/2} Z \left( \frac{|x|}{tN + \frac{1}{N}} \right) , \end{aligned}$$

where *Z* decays super-polynomially.

#### Proof

This is immediate on applying Poisson summation followed by stationary phase. $\square$

### The one-dimensional case

As in, for example, equation (8) of Bourgain-Demeter [3], we have:

#### Lemma 3.2

(Weyl bound) If $a\in {\mathbb {Z}} \setminus \{0\}$, $q \in \{ 1,\dots , N \}$, $(a,q)=1$ and $\left| t-\frac{a}{q}\right| \le \frac{1}{q N}$:

3.1 $$\begin{aligned} \forall y \in {\mathbb {R}}, \qquad \left| K^{(1)}_N(t,y) \right| \lesssim _\epsilon \frac{N^{1+\epsilon }}{\sqrt{q}( 1 + N | t - \frac{a}{q} |^{1/2})} \lesssim \frac{N^{1+\epsilon }}{\sqrt{q}}. \end{aligned}$$

We now define a decomposition into major and minor arcs: for *Q* a power of 2, $c_0$ a constant which will be chosen small enough, and $Q \le c_0 N$,

$$\begin{aligned} \Lambda _Q(t) = \sum _{\begin{array}{c} q \in {\mathbb {N}} \\ \frac{1}{2} Q \le q < Q \end{array}} \sum _{\begin{array}{c} a \in {\mathbb {Z}}^* \\ (a,q) = 1 \end{array}} \textbf{1}_{[-1,1]} \left( NQ\left( t-\frac{a}{q} \right) \right) . \end{aligned}$$

In the definition of $\Lambda _Q$, the integer *a* is not allowed to be zero; it turns out to be convenient to single out the case $a=0$, by letting

$$\begin{aligned} \Lambda _0(t) = \textbf{1}_{[-\frac{1}{N},\frac{1}{N}]}. \end{aligned}$$

Observe that functions of the type $\textbf{1}_{[-1,1]} \left( NQ\left( t-\frac{a}{q} \right) \right)$, with $\frac{1}{2} Q_1 \le q_1 \le Q_1 \le c_0 N$ and $\frac{1}{2} Q_2 \le q_2 \le Q_2 \le c_0 N$ are disjoint if $(a_1,q_1) = (a_2,q_2) =1$ and $\frac{a_1}{q_1} \ne \frac{a_2}{q_2}$. Indeed, if $Q_1 \ge Q_2$,

$$\begin{aligned} \left| \frac{a_1}{q_1} - \frac{a_2}{q_2} \right| \ge \frac{1}{q_1 q_2} \ge \frac{1}{Q_1 Q_2} \ge \frac{1}{c_0 N Q_2} \ge \frac{2}{NQ_2} \ge \frac{1}{NQ_1} + \frac{1}{NQ_2}. \end{aligned}$$

Therefore, $$\Lambda _0 + \sum _{\begin{array}{c} Q \in 2^{\mathbb {N}} \\ Q < c_0 N \end{array}} \Lambda _Q$$ is the characteristic function of a set: the major acs.

The minor arcs will be the complement, with characteristic function $\rho$. This gives the decomposition, for any $t \in {\mathbb {R}}$,

3.2 $$\begin{aligned} 1 = {\Lambda _0(t)+}\sum _{\begin{array}{c} Q \in 2^{\mathbb {N}} \\ Q < c_0 N \end{array}} \Lambda _Q(t) + \rho (t). \end{aligned}$$

On the support of each of the summands above, the following bounds are available:

- On the support of $\Lambda _0$, there holds $|t| \le \frac{1}{N}$, and we resort to the short time bound.
- If *t* belongs to the support of $\Lambda _{Q}$, there exists $q \in [\frac{Q}{2},Q]$ such that $|t-\frac{a}{q}| < \frac{1}{qN}$ and Weyl’s bound gives $\displaystyle |K_N^{(1)}(t,y)| \lesssim _\epsilon \frac{N^{1+\epsilon }}{\sqrt{Q}}$.
- On the support of $\rho$, by Dirichlet’s approximation theorem, there exists $a \in {\mathbb {Z}}$ and $q \in \{1,\dots ,N\}$ relatively prime such that $|t-\frac{a}{q}| < \frac{1}{qN}$. If $q \sim N$, Weyl’s bound gives $\displaystyle |K_N^{(1)}(t,y)| \lesssim _\epsilon N^{1/2+\epsilon }$. If $q \ll N$, then, since *t* does not belong to the support of $\sum _{Q \le c_0 N} \Lambda _Q$, there holds $|t-\frac{a}{q}| \sim \frac{1}{qN}$, which implies again, by Weyl’s bound, $\displaystyle |K_N^{(1)}(t,y)| \lesssim _\epsilon N^{1/2+\epsilon }$.

### The case of generic rectangular tori

In this subsection, we assume that the tori are rectangular, or, equivalently, that the quadratic form ${\mathcal {Q}}$ is diagonal. First of all, we learn from the bounds on $K_N^{(1)}$ that, on the support of $\Lambda _{Q_1}(\beta _1 t) \dots \Lambda _{Q_k}(\beta _k t) \rho (\beta _{k+1} t) \dots \rho (\beta _d t)$, with all $Q_i \in 2^{\mathbb {N}}$ and $Q_i \le c_0 N$,

3.3 $$\begin{aligned} |K_N(t,x)| = |K_N^{(1)}(\beta _1t,x) \dots K_N^{(1)}(\beta _dt,x)| \lesssim _\epsilon \frac{N^{\frac{k+d}{2}+\epsilon }}{\sqrt{Q_1 \dots Q_k}}. \end{aligned}$$

#### Lemma 3.3

Let $\kappa , \epsilon >0$; then for generic $\beta _1, \dots , \beta _d \in [1,2]^d$ there holds, for $1 \le k \le d$, $0\le T \le N^\kappa$, and $\epsilon >0$, and for all $Q_i, N$ equal to powers of 2, with $Q_i \le N$,

$$\begin{aligned} \int _0^T \Lambda _{Q_1}(\beta _1 t) \Lambda _{Q_2}(\beta _2 t) \dots \Lambda _{Q_k}(\beta _k t)\,dt \lesssim _{\epsilon ,\kappa ,\beta _1 \dots \beta _d} N^\epsilon \frac{Q_1 \dots Q_k}{N^k} T. \end{aligned}$$

#### Proof

Without loss of generality, we can choose $\beta _1 = 1$. Indeed, if $\beta _1,\dots ,\beta _d,\frac{T}{\gamma }$ is changed to $\gamma \beta _1, \dots , \gamma \beta _d,T$, with $\gamma > 0$, the integral in the statement of the lemma changes by the factor $\gamma$. We claim that it suffices to prove that

$$\begin{aligned} \int _{1}^{2} \dots \int _{1}^{2} \int _0^T \Lambda _{Q_1}(t) \Lambda _{Q_2}(\beta _2 t) \dots \Lambda _{Q_k}(\beta _k t)\,dt \,d\beta _2 \dots d\beta _k \lesssim {\textbf{1}_{t\ge \frac{1}{4N}}} \frac{Q_1 \dots Q_k}{N^{k}} T . \end{aligned}$$

Indeed the case $t<\frac{1}{4N}$ of the lemma is then immediate, and the remaining case $\frac{1}{4N}\le t \le N^\kappa$ follows by the Borel-Cantelli lemma as explained in Appendix A. By definition of $\Lambda _Q$,

$$\begin{aligned} \int _{1}^{2} \Lambda _Q(\beta t) \,d\beta = \frac{1}{t} \int _{t}^{2t} \Lambda _Q(y)\,dy = \frac{1}{t} \sum _{\begin{array}{c} (a,q) = 1 \\ \frac{1}{2}Q<q<Q \end{array}}\int _t^{2t} \textbf{1}_{[-1,1]}\left( NQ \left( y-\frac{a}{q} \right) \right) \,dy. \end{aligned}$$

To estimate this integral, we observe first that, if $[t,2t] \cap [\frac{a}{q} - \frac{1}{NQ}, \frac{a}{q} + \frac{1}{NQ}] \ne \emptyset$, then

$$\begin{aligned} 2t \ge \frac{a}{q} - \frac{1}{NQ} \ge \frac{1}{Q} \left[ 1 - \frac{1}{N} \right] , \end{aligned}$$

so that, in particular $t {{}\ge \frac{1}{4Q}}$. Similarly, one can show that the number of *a*’s such that $[t,2t] \cap [\frac{a}{q} - \frac{1}{NQ}, \frac{a}{q} + \frac{1}{NQ}] \ne \emptyset$ is $\lesssim Qt$. Since furthermore the number of *q*’s in $[\frac{1}{2}Q,Q]$ is $\le Q$, and since finally the integral of $\textbf{1}_{[-1,1]}\left( NQ \left( y-\frac{a}{q} \right) \right)$ is $\lesssim \frac{1}{NQ}$, we obtain the estimate

$$\begin{aligned} \int _{1}^{2} \Lambda _Q(\beta t) \,d\beta \lesssim \frac{1}{t} \cdot Qt \cdot Q \cdot \frac{1}{NQ}{\textbf{1}_{t\ge \frac{1}{4Q}}} \lesssim \frac{Q}{N}{\textbf{1}_{t\ge \frac{1}{4Q}}}. \end{aligned}$$

Then, by Fubini’s theorem

$$\begin{aligned}&\int _{1}^{2} \dots \int _{1}^{2} \int _0^T \Lambda _{Q_1}(t) \Lambda _{Q_2}(\beta _2 t) \dots \Lambda _{Q_k}(\beta _k t)\,dt \,d\beta _2 \dots d\beta _k \\&\qquad = \int _0^T \Lambda _{Q_1}(t) \left[ \int _{1}^{2} \Lambda _{Q_2}(\beta _2 t) \,d\beta _2 \right] \dots \left[ \int _{1}^{2} \Lambda _{Q_k}(\beta _k t) \,d\beta _k \right] \,dt \\&\qquad \lesssim {\int _0^T} \Lambda _{Q_1}(t) \frac{Q_2 \dots Q_k}{N^{k-1}}\,dt \lesssim {\textbf{1}_{t\ge \frac{1}{4Q_1}}} \frac{Q_1 \dots Q_k}{N^k} T. \end{aligned}$$

$$\square $$

A consequence of this lemma is an $L^1_t L^\infty _x$ estimate on $K_N$.

#### Lemma 3.4

(Square root cancellation in $L^1_t L^\infty _x$) Let $\kappa ,\epsilon >0$; then for generic $\beta _1, \dots , \beta _d \in [1,2]^d$ there holds, for *N* a power of 2 and $\frac{1}{N}< T < N^\kappa$,

$$\begin{aligned} \frac{1}{T} \int _{1/N}^{T} \Vert K_N(t,\cdot ) \Vert _{L^\infty } \,dt \lesssim _{\epsilon ,\kappa ,\beta _1 \dots \beta _d} N^{\frac{d}{2} + \epsilon }. \end{aligned}$$

#### Proof

The first step is to use the decomposition (3.2) in each of the variables $\beta _1 t,\dots \beta _d t$. Note that, since $t > \frac{1}{N}$ and $\beta _i \ge 1$, the term $\Lambda _0(\beta _i t)$ is always zero. By the inequality (3.3), for almost any choice of $\beta _1,\dots ,\beta _d$, there holds

$$\begin{aligned}&\frac{1}{T} \int _{1/N}^{T} \Vert K_N(t,\cdot ) \Vert _{L^\infty } \,dt \\&{\qquad \lesssim _\epsilon \sum _{k=0}^d \sum _{\begin{array}{c} Q_1, \dots Q_k \le c_0 N \\ Q_i \in 2^{\mathbb {N}} \end{array}} \frac{1}{T} \int _{1/N}^T \Lambda _{Q_1}(\beta _1 t) \dots \Lambda _{Q_k} (\beta _k t) \rho (\beta _{k+1} t) \dots \rho (\beta _d t) \,dt \frac{N^{\frac{k+d}{2}+\epsilon }}{\sqrt{Q_1 \dots Q_k}}} \\&{\qquad \lesssim _{\epsilon ,\kappa ,\beta _1 \dots \beta _d} N^\epsilon \sum _{k=0}^d \sum _{Q_1, \dots Q_k \le c_0 N} \frac{Q_1 \dots Q_k}{N^k} \frac{N^{\frac{k+d}{2}}}{\sqrt{Q_1 \dots Q_k}}} \\&\qquad \lesssim N^{\frac{d}{2} + \epsilon }. \end{aligned}$$

$$\square $$

### The case of generic tori

We start with an averaging lemma.

#### Lemma 3.5

For $$A \in {\mathbb {R}}$$, $$\lambda >0$$, and $$N \ge 10$$,

$$\begin{aligned} \int _{-1}^1 \min \left( \frac{1}{\Vert \lambda h + A \Vert } , N \right) \,dh \lesssim \left\{ \begin{array}{ll} \log N &{} \text {if}\ \lambda \ge 1 \\ \frac{\textrm{log} N}{\lambda } &{} \text {if}\ \lambda \le 1, \end{array}\right. \end{aligned}$$

where, for a real number *x*, the notation $\Vert x \Vert$ stands for $\textrm{dist}(x,{\mathbb {Z}}){=\min _{k\in {\mathbb {Z}}}|k-x|}$.

#### Proof

If $\lambda \ge 1$, the left-hand side can be bounded by the average of the function $\min \left( \frac{1}{\Vert h \Vert } , N\right)$, which equals $\log N$. If $\lambda \le 1$, the left-hand side is bounded by $\int _{-1}^1 \min \left( \frac{1}{\Vert \lambda h \Vert } , N \right) \,dh \lesssim \frac{\textrm{log} N}{\lambda }$. $\square$

Armed with this lemma, we can now prove the desired square root cancellation result - its proof already appeared in [8], but we include an equivalent version here for the reader’s convenience. Recall that the measure on nonsingular symmetric matrices we consider is given by $B = \textrm{Id} + h_{ij}$, where *h* is a symmetric matrix, all of whose coefficients are independent (besides the symmetry assumption) and uniformly distributed in $\left[ \frac{1}{10d^2} , \frac{1}{10d^2} \right]$.

#### Lemma 3.6

(Square root cancellation in $L^1_t L^\infty _x$) Let $\kappa ,\epsilon >0$; then for generic $(\beta _{i,j})$, there holds, for *N* a power of 2 and $\frac{1}{N}< T < N^\kappa$,

$$\begin{aligned} \frac{1}{T} \int _{1/N}^{T} \Vert K_N(t,\cdot ) \Vert _{L^\infty } \,dt \lesssim _{\epsilon ,\kappa ,\beta _{ij}} N^{\frac{d}{2} + \epsilon }. \end{aligned}$$

#### Proof

By the Borel-Cantelli argument in Appendix A, the result would follow from the bound

$$\begin{aligned} \int \frac{1}{T} \int _{1/N}^{T} \Vert K_N(t,\cdot ) \Vert _{L^\infty } \,dt \,dB \lesssim _{\epsilon } N^{\frac{d}{2} + \epsilon }, \end{aligned}$$

which will now be proved. For $x \in {\mathbb {T}}^d$ and $t \in {\mathbb {R}}$, applying Weyl differencing gives

$$\begin{aligned} |K_N(t,x)|^2 = \sum _{m,n} \phi \left( \frac{m_1 + n_1}{2N} \right) \dots \phi \left( \frac{m_d + n_d}{2N} \right) \left( \frac{m_1 - n_1}{2N} \right) \dots \phi \left( \frac{m_d - n_d}{2N} \right) e^{2itQ(m,n)}, \end{aligned}$$

(where the sum is implicitly restricted to $m_i$, $n_i$ having the same parity). By Abel summation, this implies that

$$\begin{aligned} \Vert K_N(t,\cdot ) \Vert _{L^\infty _x} \lesssim \sum _{|n| \lesssim N} \prod _{i=1}^d \min \left( \frac{1}{\Vert t Q_i(n) \Vert } , N \right) , \end{aligned}$$

where $Q_i(n) = \sum _j \beta _{ij} n_j$. Combining the Cauchy-Schwarz inequality with the above yields

$$\begin{aligned} \int _{1/N}^{T} \Vert K_N(t,\cdot ) \Vert _{L^\infty } \,dt \,dB&\lesssim \sqrt{T} \left[ \int \int _{1/N}^{T} \Vert K_N(t,\cdot ) \Vert _{L^\infty _x}^2 \,dt \,dB \right] ^{1/2} \\&\lesssim \sqrt{T} \left[ \int \int _{1/N}^{T} \sum _{|n| \lesssim N} \prod _{i=1}^d \min \left( \frac{1}{\Vert t Q_i(n) \Vert } , N \right) \, dt \, dB \right] ^{1/2}, \end{aligned}$$

where $dB = \prod _{i \le j} d\beta _{i,j}.$ We now exchange the order of summation and integration, performing first the integration over *B*. Without loss of generality, assume that $|n_1| \sim |n|$. Note that $tQ_{1,1} (n) = t \sum _j \beta _{1,j} n_j$; therefore, by Lemma 3.4, integrating first $\int \min \left( \frac{1}{\Vert t Q_1(n) \Vert } , N \right) \,d\beta _{1,1}$ gives $\log N \langle \frac{1}{t|n|} \rangle$. We integrate next $\int \min \left( \frac{1}{\Vert t Q_2(n) \Vert } , N \right) \,d\beta _{1,2}$, giving the same result, and similarly $\int \min \left( \frac{1}{\Vert t Q_k(n) \Vert } , N \right) \,d\beta _{1,k}$, for $3 \le k \le d$. Finally, $\int \prod _{2 \le i \le j} d\beta _{i,j}$ gives *O*(1). Coming back to the sequence of inequalities above,

$$\begin{aligned} \int _{1/N}^{T} \Vert K_N(t,\cdot ) \Vert _{L^\infty } \,dt \,dB&\lesssim \sqrt{T} \left[ \sum _{|n| \lesssim N} ( \log N)^d \int _{1/N}^T \langle \frac{1}{t|n|} \rangle ^d \,dt \right] ^{1/2} \\&\lesssim T N^{d/2} (\log N)^{d/2}. \end{aligned}$$

$$\square $$

## Proof of Theorem 1.2

An important element of the proof is the optimal $L^{2} \rightarrow L^{p_{ST}}$ bound for spectral projectors. As observed in the previous article by the authors [10], it is a consequence of the $\ell ^2$ decoupling bound of Bourgain-Demeter. The statement is as follows.

### Theorem 4.1

If $\lambda > 1$ and $\delta <1$, for any positive definite quadratic form ${\mathcal {Q}}$,

$$\begin{aligned} \Vert P_{\lambda ,\delta } \Vert _{L^2 \rightarrow L^{p_{ST}}} \lesssim (1 + \lambda \delta )^{\frac{1}{p_{ST}}}. \end{aligned}$$

We now turn to the proof of Theorem 1.2.

### Proof

Step 1: Allowing more general cutoff functions. Define the spectral projector

$$\begin{aligned} P'_{\lambda ,\delta } = \zeta \left( \frac{{\mathcal {Q}}(\nabla ) + \lambda ^2}{\lambda \delta } \right) , \end{aligned}$$

where $\zeta$ is a Schwartz function such that $\zeta (0) > 0$ and $\widehat{\zeta }$ is compactly supported.

We claim that it suffices to prove Theorem 1.2 for the spectral projector $P'_{\lambda ,\delta }$ instead of $P'_{\lambda ,\delta }$. Indeed, assume that $P'_{\lambda ,\delta }$ enjoys the bound in this theorem. Since there exists $c>0$ such that $\zeta (x) \ge c \textbf{1}_{[-c,c](x)}$, the desired bound follows^2^ for the operator $\textbf{1}_{[-c,c]} \left( \frac{{\mathcal {Q}}(\nabla ) + \lambda ^2}{\lambda \delta } \right)$. This implies in turn this bound for the operator $\textbf{1}_{[-a,a]} \left( \frac{\sqrt{-{\mathcal {Q}}(\nabla )} - \lambda }{\delta } \right)$, for a constant $a>0$. Finally, this implies the desired bound for $P_{\lambda ,\delta }$ since $|\chi (x)|$ can be bounded by a finite sum of translates of $\textbf{1}_{[-a,a]}$.

We now claim that $P'_{\lambda ,1}$ enjoys the Sogge bounds (1.3), just like $P_{\lambda ,1}$. This follows from writing

$$\begin{aligned} P_{\lambda ,1}' = \sum _{n > -\frac{\delta }{\lambda }} P'_{\lambda ,1} \textbf{1}_{[\lambda +(n-1)\delta , \lambda + n\delta ]} \left( \sqrt{-{\mathcal {Q}}(\nabla )} \right) \end{aligned}$$

and bounding

$$\begin{aligned} \Vert P_{\lambda ,1}' \Vert _{L^2 \rightarrow L^p}\lesssim & {} c_n \sum _{n > -\frac{\delta }{\lambda }} \left\| \textbf{1}_{[\lambda +(n-1)\delta , \lambda + n\delta ]} \left( \sqrt{-{\mathcal {Q}}(\nabla )} \right) \right\| _{L^2 \rightarrow L^p} \;\; \text {with} \;\; c_n \\= & {} \sup _{(n-1)\delta< x < n\delta } \left| \zeta \left( \frac{-x^2 + \lambda ^2}{\lambda \delta } \right) \right| . \end{aligned}$$

The rapid decay of $\zeta$ implies that $|c_n| \lesssim n^{-N}$ for any *N*, while $\textbf{1}_{[\lambda +(n-1)\delta , \lambda + n\delta ]}$ enjoys the Sogge bounds. Thus, it is not hard to sum the above series, and deduce that $P_{\lambda ,1}'$ also enjoys the Sogge bounds.

By a similar argument, it can be shown that Theorem 4.1 applies to $P'_{\lambda ,1}$.

Step 2: splitting the spectral projector. Writing the function $x \mapsto \zeta \left( \frac{{\mathcal {Q}}(x) + \lambda ^2}{\lambda \delta } \right)$ as a Fourier transform, the operator $P_{\lambda ,\delta }'$ becomes

4.1 $$\begin{aligned} P_{\lambda ,\delta }' = \int _{{\mathbb {R}}} \lambda \delta \widehat{\zeta }(\lambda \delta t) e^{-2\pi i \lambda ^2 t} e^{-2\pi it{\mathcal {Q}}(\nabla )} \,dt, \end{aligned}$$

with the kernel

$$\begin{aligned} P_{\lambda ,\delta }'(x) = \int _{{\mathbb {R}}} \lambda \delta \widehat{\zeta }(\lambda \delta t) e^{-2\pi i \lambda ^2 t} K_N(t,x) \,dt; \end{aligned}$$

here, we choose *N* to be a power of 2 in the range $[2\lambda ,4\lambda ]$.

The basic idea is to split the integral giving $P_{\lambda ,\delta }$ into two pieces, $|t| < \lambda ^{-1}$ and $|t| > \lambda ^{-1}$. The former corresponds to an operator of the type $\delta P_{\lambda ,1}'$, for which bounds are well-known: this corresponds to the classical Sogge theorem. The latter can be thought of as an error term, it will be bounded by interpolation between $p=p_{ST}$ and $p= \infty$, and it is for this term that genericity is used.

Turning to the implementation of this plan, we write

$$\begin{aligned} P_{\lambda ,\delta }'&= \int _{{\mathbb {R}}} \lambda \delta \widehat{\zeta }(\lambda t) \widehat{\zeta }(\lambda \delta t) e^{-2\pi i \lambda ^2 t} e^{-2\pi it {\mathcal {Q}}(\nabla )} \,dt \\&\quad +\int _{{\mathbb {R}}} \lambda \delta [1- \widehat{\zeta }(\lambda t)] \widehat{\zeta }(\lambda \delta t) e^{-2\pi i \lambda ^2 t} e^{-2\pi it{\mathcal {Q}}(\nabla )} \,dt \\&= P^{\textrm{small}}_{\lambda ,\delta } + P^{\textrm{large}}_{\lambda ,\delta } \end{aligned}$$

$\underline{\text {Step 3: Bounding the term corresponding to small}\ t.}$ Observe that $P_{\lambda ,\delta }^{\textrm{small}}$ can be written $\delta P''_{\lambda , 1}$, where $P''_{\lambda , 1}$ is a variation on $P'_{\lambda ,1}$; this can be compared to the definition of $P_{\lambda ,\delta }^{\textrm{small}}$ and (4.1). We saw in Step 1 that $P'_{\lambda ,1}$ enjoys the Sogge bounds, and this remains true for $P''_{\lambda ,1}$.

Furthermore, by a classical $TT^*$ argument, the operator norm of the spectral projector $L^{p'} \rightarrow L^p$ is the square of the operator norm of the spectral projector $L^2 \rightarrow L^p$ (once again, up to redefining the cutoff function $\chi$).

Therefore, it enjoys the bound

4.2 $$\begin{aligned} {\Vert P_{\lambda ,\delta }^{\textrm{small}} \Vert _{L^{p'} \rightarrow L^p} \lesssim \delta \Vert P''_{\lambda , 1} \Vert ^2_{L^2 \rightarrow L^p} \lesssim \lambda ^{d-1-\frac{2d}{p}} \delta \qquad \text {for}\ p \ge p_{ST}.} \end{aligned}$$

$\underline{\text {Step 4: Bounding the term corresponding to large}\ t.}$ In order to bound this term, we will interpolate between

- The case $p=p_{ST}$: in this case, we resort to Theorem 4.1. We saw in Step 1 that it applies to $P'_{\lambda ,\delta }$, and, by the same argument, it applies to $P''_{\lambda ,\delta }$. This gives
  $$\begin{aligned} \Vert P_{\lambda ,\delta }^{\textrm{large}} \Vert _{L^{p_{ST}'} \rightarrow L^p_{ST}}&\lesssim \Vert P'_{\lambda ,\delta } \Vert _{L^{p_{ST}'} \rightarrow L^p_{ST}} + \Vert P^{\textrm{small}}_{\lambda ,\delta } \Vert _{L^{p_{ST}'} \rightarrow L^p_{ST}} \\&\lesssim \Vert P'_{\lambda ,\delta } \Vert _{L^{p'_{ST}} \rightarrow L^{p_{ST}}} +\delta \Vert P''_{\lambda ,\delta } \Vert _{L^{p'_{ST}} \rightarrow L^{p_{ST}}} \\&\lesssim _\epsilon \lambda ^\epsilon [1 + (\lambda \delta )^{2/p_{ST}} + \delta \lambda ^{2/p_{ST}}] \lesssim \lambda ^\epsilon [ 1 + (\lambda \delta )^{2/p_{ST}}]. \end{aligned}$$
- The case $p = \infty$: in this case, we resort to Lemma 3.4 (generic rectangular tori) and Lemma 3.6 (generic tori). In order for these lemmas to apply, we add a further requirement on $\zeta$, namely that its Fourier transform be 1 in a neighbourhood of zero. Then, for almost any choice of $(\beta _{ij})$,
  $$\begin{aligned} \Vert P_{\lambda ,\delta }^{\textrm{large}} \Vert _{L^1 \rightarrow L^\infty } \lesssim \int _{{\mathbb {R}}} \lambda \delta |1 - \widehat{\zeta }(\lambda t) | \left| \widehat{\zeta }(\lambda \delta t)\right| \Vert K_{N} (t,\cdot ) \Vert _{L^\infty } \,dt \lesssim _{\beta ,\epsilon } \lambda ^{\frac{d}{2} + \epsilon }. \end{aligned}$$

Interpolating between these two bounds gives for almost any choice of $(\beta _{ij})$,

4.3 $$\begin{aligned} \Vert P_{\lambda ,\delta }^{\textrm{large}} \Vert _{L^{p'} \rightarrow L^p} \lesssim _{\beta ,\epsilon } (1 + \lambda \delta )^{2/p} \lambda ^{\frac{d}{2} \left( 1 - \frac{p_{ST}}{p} \right) + \epsilon } \qquad \text {for}\ p \ge p_{ST}. \end{aligned}$$

Step 5: conclusion. Finally, combining (4.2) and (4.3), and using that $\Vert P_{\lambda ,\delta }\Vert _{L^2 \rightarrow L^p}^2 \sim \Vert P_{\lambda ,\delta }^2 \Vert _{L^{p'} \rightarrow L^p}$ (by the classical $TT^*$ argument) gives

$$\begin{aligned} \Vert P_{\lambda ,\delta }\Vert _{L^2 \rightarrow L^p} \lesssim _{\beta ,\epsilon } \lambda ^{\frac{d-1}{2} - \frac{d}{p}} \delta ^{1/2} + (1 + \lambda \delta )^{\frac{1}{p}} \lambda ^{\frac{d}{4} \left( 1 - \frac{p_{ST}}{p} \right) + \epsilon }, \end{aligned}$$

from which the desired result follows. $\square$

## Some linear algebra

In this section we assemble technical tools to attack Problem 1.4. Recall that the goal is to count the number of matrices

$$\begin{aligned} \left( \begin{matrix} m^2_{11}&{}\cdots &{}m^2_{1b}\\ \vdots &{}\ddots &{}\vdots \\ m^2_{d1}&{}\cdots &{}m^2_{db} \\ \lambda ^2&{}\cdots &{}\lambda ^2 \end{matrix}\right) , \end{aligned}$$

where the $m_{ij}$ are integers in given dyadic intervals and the maximal subdeterminants of *P* also lie in some specified dyadic intervals. The idea is to add the columns one by one, so that we count the number of possible $(m_{11},\dotsc ,m_{d1})$, and for each possibility we count the number of $(m_{12},\dotsc ,m_{d2})$, and so on. The main goal of this section is Lemma 5.7, which can be understood as an estimate for the *measure* of the *real* vectors $(m_{1k},\dotsc ,m_{dk})$ which are within a distance *O*(1) of a vector satisfying the required conditions, given the previous columns.

In this and the next section we will often use the notation $D_k(M), D_k^{(\ell )}(M)$ defined in Sect. 2.

### Singular values and largest subdeterminants

We begin with a number of general statements about the size of the subdeterminants of a $p\times q$ matrix, and their relation to the *singular value decomposition*, a type of canonical form for matrices. Throughout this subsection, implicit constants in $\lesssim$ and $\sim$ notation may depend on *p* and *q*.

#### Lemma 5.1

(Singular value decomposition) Let $M \in {\mathbb {R}}^{p\times q}$ and let $m=\min (p,q)$. Then there are $U\in O(p)$, $V\in O(q)$ and (uniquely defined) *singular values* $\sigma _1(M) \ge \cdots \ge \sigma _{m}(M)\ge 0$ such that

5.1 $$\begin{aligned} M = U \begin{pmatrix} \Sigma \\ 0 \end{pmatrix} V \quad \text {or}\quad U \begin{pmatrix} \Sigma \; 0 \end{pmatrix} V, \quad \text {where} \quad \Sigma = \textrm{diag}( \sigma _1(M), \dots , \sigma _m(M)), \end{aligned}$$

and where 0 is a matrix of zeroes (possibly empty).

#### Lemma 5.2

(Stability of $\sigma _k$ and $D_k$ under multiplication by orthogonal matrices)] If $k \le \min (p,q)$ and *M* is a matrix in ${\mathbb {R}}^{p\times q}$,

(i) If $U\in O(p)$, then $D_k(UM) \sim D_k(M)$ and $\sigma _k(UM) = \sigma _k(M)$.
(ii) If $U\in O(q)$, then $D_k(MU) \sim D_k(M)$ and $\sigma _k(MU) = \sigma _k(M)$.

#### Proof

The statements (*i*) and (*ii*) are symmetric, so that we will only focus on (*i*). Let ${\mathcal {I}}\subset \{1,\dotsc , p\},{\mathcal {J}}\subset \{1,\dotsc ,q\}, \#{\mathcal {I}}=\#{\mathcal {J}}=k$. The Cauchy-Binet identity gives

$$\begin{aligned} \det \, (UM)_{i \in {\mathcal {I}},j\in {\mathcal {J}}} = \det \, U_{i \in {\mathcal {I}}, \ell } \cdot M_{\ell , j\in {\mathcal {J}}} =\sum _{\begin{array}{c} {\mathcal {K}}\subset \{1,\dotsc ,p\}\\ \#{\mathcal {K}} = k \end{array}} \det \, (U_{i\ell })_{i\in {\mathcal {I}},\ell \in {\mathcal {K}}} \det \, (M_{\ell j})_{\ell \in {\mathcal {K}},j\in {\mathcal {J}}}. \end{aligned}$$

Hence $D_k(UM)\lesssim D_k(M)$ and repeating the argument with $U^{-1}M$ in place of *M* shows that $D_k(M)\lesssim D_k(UM)$ as well.

Finally, it follows from the uniqueness of the $(\sigma _i)$ in (5.1) that $\sigma _k(UM) = \sigma _k(M)$.

$$\square $$

#### Corollary

(Relation between the $D_k$ and $\sigma _k$) If $k \le \min (p,q)$, the singular values and the maximal subdeterminants are such that

$$\begin{aligned} \sigma _k(M) \sim D_{k-1}(M)^{-1}D_k(M), \end{aligned}$$

where we use the convention that $0^{-1} 0=0$, or equivalently

$$\begin{aligned} D_k(M) \sim \sigma _1(M) \dots \sigma _k(M). \end{aligned}$$

#### Proof

By lemmas 5.1 and 5.2, it suffices to prove these formulas for a rectangular diagonal matrix; but then they are obvious. $\square$

#### Lemma 5.3

Given a matrix $M \in {\mathbb {R}}^{p\times q}$, we can change the order of its columns so that for each $k\le \ell \le \min (p,q)$,

5.2 $$\begin{aligned} D^{(\ell )}_k(M) \sim D_k(M). \end{aligned}$$

#### Proof

We claim first that it suffices to prove the result for the matrix *UM*, where *U* is orthogonal. Indeed, denoting $M^{(\ell )}$ for the restriction of *M* to its first $\ell$ columns, this implies, in combination with Lemma 5.2,

$$\begin{aligned} D_k^{(\ell )}(M) = D_k(M^{(\ell )}) \sim D_k(UM^{(\ell )}) =D_k^{(\ell )}(UM) \sim D_k(UM) \sim D_k(M), \end{aligned}$$

which is the desired result. For the remainder of the proof, we write for simplicity $\sigma _i=\sigma _i(M)$.

We can choose *U* as in Lemma 5.1, in which case, assuming for instance $p \ge q$, it suffices to deal with the case

$$\begin{aligned} M = \left( \sigma _1 L_1, \dots \sigma _q L_q, 0, \dots 0 \right) ^T, \end{aligned}$$

where $L_i \cdot L_j = \delta _{ij}$. The 0 entries are irrelevant, so we can assume that

$$\begin{aligned} M = \left( \sigma _1 L_1, \dots \sigma _q L_q \right) ^T = (M_{ij})_{1 \le i,j \le q}. \end{aligned}$$

We now claim that, after permuting the columns of *M*, it can be ensured that, for any *k*, the top left square matrix of dimension $k \times k$ has nearly maximal subdeterminant:

5.3 $$\begin{aligned} \det (M_{ij})_{1 \le i,j \le k} \sim D_k(M). \end{aligned}$$

The construction of the matrix permutation is iterative and proceeds as follows: expanding the determinant of *M* with respect to the last row, we see that

$$\begin{aligned} \sigma _1 \dots \sigma _q = \textrm{det} M = {\sum _{i=1}^p} (-1)^{q+i} M_{q,i} \textrm{det} M^{\{q,i\}}, \end{aligned}$$

where $M^{\{q,i\}}$ is the matrix obtained from *M* by removing the *q*-th row and the *i*-th column. Since $|M_{q,i}| \le \sigma _q$ and $\textrm{det} M^{\{q,i\}} \lesssim \sigma _{1} \dots \sigma _{q-1}$ for all *i*, we can find $i_0$ such that $|M_{q,i_0}| \sim \sigma _q$, and $\textrm{det} M^{\{q,i_0\}} \sim \sigma _{1} \dots \sigma _{q-1}$. Exchanging the columns $i_0$ and *q*, the resulting matrix satisfies (5.3) for $k = q-1$.

We now consider the matrix $N = (M_{ij})_{1 \le i,j \le q-1}$, which was denoted $M^{\{q,i_0\}}$ before columns were permuted. It is such that entries in the last row are $\le \sigma _{q-1}$, and subdeterminants of size $q-2$ are bounded by $\sigma _1 \dots \sigma _{q-2}$. Therefore, the same argument as above can be applied, and it proves (5.3) for $k = q-2$. An obvious induction leads to the desired statement. $\square$

### Describing some convex bodies

We can use the subdeterminants studied above to describe certain convex bodies. Our first result concerns the measure of a neighbourhood of a convex hull.

#### Lemma 5.4

Let *X* denote the $d \times d$ matrix with columns $x^{(i)}$. Then

$$\begin{aligned} \textrm{mes}\left\{ \sum _{i=1}^d t_i {x}^{(i)}+ {w}, \, |t_i|\le 1, \, |{w}|\le 1\right\} \lesssim 1+ \sum _{k=1}^d D_k(X) {.} \end{aligned}$$

#### Proof

Let *M* be the $d \times 2d$ matrix $(X | \textrm{Id})$, so that the set whose measure we want to estimate can be written (up to a multiplicative constant) *MB*(0, 1). By Lemma 5.1 (singular value decomposition), we can write $M = U (\Sigma | 0) V$; then

$$\begin{aligned} \textrm{mes}M B(0,1)= & {} \textrm{mes}U (\Sigma | 0) V B(0,1) \\= & {} \textrm{mes}(\Sigma | 0) B(0,1) \lesssim \textrm{mes}\Sigma B(0,1) = \det \Sigma \sim D_d(M). \end{aligned}$$

There remains to evaluate $D_d(M)$; owing to the specific structure of *M*,

$$\begin{aligned} D_d(M) = D_d( X | \textrm{Id}) \lesssim \sum _{k=1}^d D_k(X). \end{aligned}$$

$$\square $$

We can also describe a subset of a convex hull cut out by linear inequalities, showing that it is contained in a potentially smaller convex hull.

#### Lemma 5.5

Given linearly independent ${{v}^{(1)},\dotsc {v}^{(d)}} \in ({\mathbb {R}}^d)^d,$ and $Y_i>0,Z_i>0$ there are ${{w}^{(1)},\dotsc {w}^{(d)}} \in ({\mathbb {R}}^d)^d,$ with

5.4 $$\begin{aligned} w^{(i)}_j \lesssim \min (Y_i|{v}^{(i)}|,Z_j), \end{aligned}$$

such that

5.5 $$\begin{aligned} \left\{ {z}\in {\mathbb {R}}^d: {z}=\sum _{i=1}^d y_i {v}^{(i)}, \, |y_i|\le Y_i, \,|z_i|\le Z_i\right\} \subset \left\{ \sum _{i=1}^d t_i {w}^{(i)}:|t_i| \lesssim 1\right\} . \end{aligned}$$

#### Proof

Let *Y* be the matrix with columns $Y_i {v}^{(i)}$, which, without loss of generality, can be assumed to have nondecreasing norms. We claim that its singular values, $\tau _i$, are such that

5.6 $$\begin{aligned} \tau _i \lesssim Y_i |v^{(i)}|. \end{aligned}$$

Indeed, by the Courant minimax principle, $\tau _k$ can be characterized as follows

$$\begin{aligned} \tau _k&= \sigma _k (Y) = \min _{\dim E = d+1-k} \max _{\begin{array}{c} x \in E \\ | x | =1 \end{array}} | Y x| \le \max _{\begin{array}{c} x_{d-k+2} {=}\cdots = x_{d} = 0 \\ | x | =1 \end{array}} |Yx| \\&\lesssim \max _{j = 1, \dots , d-k+1} |Y_j| |v^{(j)}| = |Y_{d-k+1}| |v^{(d-k+1)}|. \end{aligned}$$

Let *Z* be the matrix $\textrm{diag}(Z_1,\dotsc ,Z_d)$, and let $M = ( Y^{-T} \,|\, Z^{-T} )^T \in {\mathbb {R}}^{2d \times d}$. Then the set on the left-hand side of (5.5) is contained in $\{ z: |Mz| \lesssim 1 \}$. By Lemma 5.1, we can write $M = U ( \Sigma ^T \,|\, 0 )^T V$, so that the set $\{ z : |Mz| \lesssim 1 \}$ can now be written (up to a multiplicative constant) as *WB*(0, 1), with

$$\begin{aligned} W = V^{-1} \Sigma ^{-1}. \end{aligned}$$

We can now define the ${w}^{(i)}$ to be the columns of *W*; in order to establish the lemma, it suffices to prove the inequality (5.4). Note first that

5.7 $$\begin{aligned} |{w}^{(i)}|\lesssim \sigma _i(M)^{-1}. \end{aligned}$$

Next, denoting $$U = \begin{pmatrix} U_1 \; U_2 \\ U_3 \; U_4 \end{pmatrix}$$ where each $U_i$ is a $d\times d$ matrix, we have $Y^{-1} = U_1\Sigma V$. Therefore,

5.8 $$\begin{aligned} (\tau _{d+1-i})^{-1} = \sigma _i(Y^{-1}) = \sigma _i(U_1 \Sigma V) \lesssim \sigma _i(\Sigma ) = \sigma _i(M). \end{aligned}$$

Combining (5.6),  (5.7) and (5.8),

$$\begin{aligned} |\vec {w}^{(i)}|\le \sigma _i(M)^{-1} \lesssim \tau _{d+1-i} \lesssim Y_i |v^{(i)}|. \end{aligned}$$

Finally, $W = Z U_3$, which gives $|w^{(i)}_j| \lesssim Z_j$. $\square$

### Extending matrices with prescribed largest subdeterminants

We now start to describe the columns which may be added to a given $p\times k$ matrix, with a prescribed effect on its singular values.

#### Lemma 5.6

Let *M* be a $p \times k$ matrix, which admits a singular value decomposition as in (5.1). For some fixed $C>0$, let

$$\begin{aligned} S(M,R)= & {} \{ x \in {\mathbb {R}}^p:D_j(M | x) \le C D_j(M) \\{} & {} \qquad (1 \le j \le \min (p,k)),\, D_{k+1}(M | x) \le R \;\;(p \ge k+1) \} \end{aligned}$$

and set

$$\begin{aligned} \tau _i = {\left\{ \begin{array}{ll} \sigma _i(M)&{}\text {if} \; i\le k, \\ \min (\sigma _k(M),\frac{R}{D_k(M)}) &{} \text {if} \; i\ge k+1 \; \text {and} \; p \ge k+1. \end{array}\right. } \end{aligned}$$

Then, denoting $U^{(i)}$ for the columns of the matrix *U* from the singular value decomposition of *M*,

$$\begin{aligned} S(M,R) \subset \left\{ \sum _{i=1}^p y_i U^{(i)} : | y_i| {\lesssim _{C,p,k}} \tau _i \right\} . \end{aligned}$$

#### Proof

In the proof we allow all implicit constants in $\lesssim ,\sim$ notation to depend on *C*, *p*, *k*.

$\underline{\text {Step 1:}\ p \ge k+1\ \text {and}\ U = \textrm{Id}}$. Then the singular value decomposition of *M* is $$M = \begin{pmatrix} \Sigma \\ 0 \end{pmatrix} V$$ and

$$\begin{aligned} (M | x) = \begin{pmatrix} \Sigma V &{} x' \\ 0 &{} x'' \end{pmatrix}, \qquad x = \begin{pmatrix} x' \\ x'' \end{pmatrix}. \end{aligned}$$

If $x \in S(M,R)$, it is immediate that $|x''| \lesssim \frac{R}{D_k(M)}$, by considering submatrices consisting of the first *k* rows, together with one of the last $p-k$ lines). Furthermore, by considering submatrices consisting of a $(k-1) \times (k-1)$ submatrix of $\Sigma V$, one of the $p-k$ last rows, and the last column, we have

$$\begin{aligned} | x''| D_{k-1}(M) \lesssim D_k(M). \end{aligned}$$

It follows that $|x''| \lesssim \sigma _k(M).$

We will now focus on the $k \times (k+1)$ matrix made up of the first *k* rows of (*M*|*x*), namely

$$\begin{aligned} \begin{pmatrix} \sigma _1(M) V^{(1)} &{} x_1 \\ \vdots &{} \vdots \\ \sigma _k(M) V^{(k)} &{} x_k \end{pmatrix} \end{aligned}$$

(where $V^{(i)}$ stands for the *i*-th row of *V*). We now prove by induction on *n* that $|x_i| \le \sigma _i(M)$ if $i \le n$; this assertion for $n=k$ is the desired result. The case $n=1$ being immediate, we can assume the assertion holds at rank *n*, and aim at proving it at rank $n+1$.

The *n* first rows of *V* are orthogonal, therefore we can delete the last $n-k$ rows and some $k-n$ columns of *V* to get an $n \times n$ matrix with a determinant $\sim 1$; denote this matrix $\widetilde{V}$ and its rows $\widetilde{V}^{(1)},\dotsc , \widetilde{V}^{(n)}$.

Note that the $n\times n$ matrix with rows $\sigma _1(M) \widetilde{V}^{(1)} ,\dotsc , \sigma _n(M) \widetilde{V}^{(n)}$ has determinant $\sim D_n(M)$.

We now consider the submatrix of *M* obtained by deleting the last $n-k-1$ rows and the same columns that were deleted from *V* to make $\widetilde{V}$. That is,

$$\begin{aligned} \widetilde{M} = \begin{pmatrix} \sigma _1(M) \widetilde{V}^{(1)} &{} x^1 \\ \vdots &{} \vdots \\ \sigma _n(M) \widetilde{V}^{(n)} &{} x^n \\ \sigma _{n+1}(M) \widetilde{V}^{(n+1)} &{} x^{n+1} \end{pmatrix}. \end{aligned}$$

We further write $\widetilde{M}^{\{i , n+1 \}}$ for the matrix $\widetilde{M}$ with *i*-th row and last column removed. Expanding the determinant of $\widetilde{M}$ with respect to the last column, we find that

$$\begin{aligned} \det (\widetilde{M}) = \sum _{i=1}^{n+1} (-1)^{i+n+1} x^i \det \widetilde{M}^{\{i , n+1 \}} + x^{n+1} \det \widetilde{M}^{\{n+1, n+1 \}}. \end{aligned}$$

By the induction assumption, $|x_i| \le \sigma _i(M)$ for $1 \le i \le n$. Furthermore, $\det \widetilde{M}^{\{i , n+1 \}} \lesssim \frac{D_{n+1}(M)}{\sigma _i(M)}$ for $1 \le i \le n$, and we saw that $\det \widetilde{M}^{\{n+1, n+1 \}} \sim D_n(M)$. Finally, the definition of *S*(*M*, *R*) requires that $\det (\widetilde{M}) \lesssim D_{n+1}(M)$. Combining these observations and the above equality implies that, if $x \in S(M,R)$,

$$\begin{aligned} |x^{n+1}| D_n(M) \lesssim D_{n+1}(M), \qquad \text {i.e.} \qquad |x^{n+1}| \lesssim \sigma _{n+1}(M). \end{aligned}$$

$\underline{\text {Step 2: general case}\ p \ge k+1.}$ Then the singular value decomposition of *M* is $$M = U \begin{pmatrix} \Sigma \\ 0 \end{pmatrix} V$$. Setting $y = U^{-1} x$, we can write

$$\begin{aligned} D_j(UDV | x) = D_j (U (DV | y) ) \sim D_j(DV|y), \qquad \text {where} \qquad D = \begin{pmatrix} \Sigma \\ 0 \end{pmatrix}. \end{aligned}$$

Then $x \in S(M,R)$ if and only if $y \in S(DV,R) \subset \{y: |y_i| \lesssim \tau _i \}$. The desired result follows for $x = Uy$.

$\underline{\text {Step 3: the case}\ p \le k.}$ Similarly to the case $p \ge k+1$, one deals first with $U = \textrm{Id}$. Then

$$\begin{aligned} M = (\Sigma | 0) V = (\Sigma V_1) \end{aligned}$$

(where $V_1$ is the $p \times k$ upper submatrix of *V*). Then

$$\begin{aligned} (M|x) = ( \Sigma V_1 | x). \end{aligned}$$

Proceeding as in Step 1, one can deduce that $|x_i| \lesssim \tau _i$ if $1 \le i \le p$, and the desired conclusion follows as in Step 2. $\square$

We can apply the last lemma to Problem 1.4, with some technical complexity coming from the constant entries in the last row of the matrix *P* appearing there. In the following lemma one should think of *M* as the first *k* columns of *P*, and *x* as being a column $(m_{1(k+1)}^2, \dotsc , m_{d(k+1)}^2, \lambda ^2)^T$ to be adjoined to the matrix *M*. As the $m_{i(k+1)}$ range over integers of size $\sim \mu _i$, the vector ${\mathcal {M}}{x} = (m_{1(k+1)}^2\mu _1^{-1}, \dotsc , m_{d(k+1)}^2\mu _d^{-1})^T$ then takes values which are separated from each other by distances $\gtrsim 1$. In Sect. 6.2 we will use this to bound the number of integral $m_{i(k+1)}$ by the measure of a neighbourhood of the permissible real vectors ${\mathcal {M}}{x}$. It is this measure that is estimated in (5.9).

#### Lemma 5.7

Adopting the notation of Lemma 5.6, let $\mu _1\ge \dotsc \ge \mu _{p-1} >0$ and let ${\mathcal {M}}$ be the $(p-1) \times p$ matrix defined by

$$\begin{aligned} {\mathcal {M}}= ( \textrm{diag}(\mu _1^{-1}, \dots , \mu _{p-1}^{-1}) | 0). \end{aligned}$$

As in Lemma 5.6 let *M* be a $p \times k$ matrix, fix $C>0$ and put

$$\begin{aligned} S(M,R)= & {} \{ x \in {\mathbb {R}}^p:D_j(M | x) \le C D_j(M) \\{} & {} \qquad (1 \le j \le \min (p,k)),\, D_{k+1}(M | x) \le R \;\;(p \ge k+1) \} , \end{aligned}$$

and let $\tau _i=\sigma _i(M)$ or $\min (\sigma _k(M),\frac{R}{D_k(M)})$ for $i\le k$ or $i>k$ respectively. Then, for any $A>0$, if $M_{p1}>\epsilon \sigma _{1}(M)$ for some $\epsilon >0$, then

5.9 $$\begin{aligned}&\textrm{mes}\left\{ {\mathcal {M}}{x}+ {w}:{x}\in S(M,R), \, x_p=A,\,{|x_i| \in \left[ \tfrac{\mu _i^2}{C}, C \mu _i^2\right] } \;(i<p) ,\, |{w}|\le 1\right\} \nonumber \\&\quad \lesssim _{\epsilon ,p,k,C} 1 + \sum _{k=1}^{p-1} D_k(\widetilde{W}), \end{aligned}$$

where $\widetilde{W}$ is a $(p-1) \times (p-1)$ matrix with entries such that

$$\begin{aligned} | \widetilde{W}_{ij} | {\lesssim _{p,k,C}} \mu _j^{-1} \min (\tau _{i+1},\mu _j^2), \end{aligned}$$

so that $$D_k(\widetilde{W}) {\lesssim _{p,k,C}} \max _{\begin{array}{c} i_1,\dotsc ,i_k\ \textrm{distinct}\\ j_1,\dotsc ,j_k\ \textrm{distinct} \end{array}} \prod _{\ell =1}^{k} \mu _{j_\ell }^{-1} \min (\tau _{i_\ell +1},\mu _{j_\ell }^2)$$.

#### Proof

In the proof we allow all implicit constants in $\lesssim ,\sim$ notation to depend on *C*, *p*, *k*. Taking the difference of two vectors in the set on the right-hand side of (5.9), we see that it suffices to prove the desired statement for $A=0$, and the condition $|x_i| \sim \mu _i^2$ replaced by $|x_i| \lesssim \mu _i^2$. In other words, it suffices to prove

$$\begin{aligned} \textrm{mes}\{{\mathcal {M}}{x}+ {w} : {x}\in S(M,R), \, x_p=0,\, |x_i| \lesssim \mu _i^2 \;(i<p) ,\, |{w}|\le 1\} \lesssim _\epsilon 1 + \sum _{k=1}^{p-1} D_k(\widetilde{W}). \end{aligned}$$

Define the projector *P* on the first $p-1$ coordinates of a vector of ${\mathbb {R}}^p$:

$$\begin{aligned} P( (x_1 ,\dots , x_p)^T) = (x_1, \dots , x_{p-1}, 0)^T. \end{aligned}$$

Let *U*, *V* be matrices as in (5.1) and let $U^{(i)}$ be the *i*th column of *U*. Since $M_{p1}>\epsilon \sigma _{1}(M)$ there is $i_0$ such that $[MV^{-1}]_{pi_0}\gtrsim \epsilon \sigma _{1}(M)$, that is to say $U^{(i_0)}_p\sigma _{i_0}(M)\gtrsim \epsilon \sigma _{1}(M)$, whence $U^{(i_0)}_p\gtrsim _\epsilon 1$ and $\sigma _{i_0}(M)\gtrsim _\epsilon \sigma _{1}(M)$.

By Lemma 5.6,

$$\begin{aligned} \left\{ x \in S(M,R) : x_p=0 \right\} \subset \left\{ x:x=\sum _{i=1}^p y_i U^{(i)} : |y_i| \lesssim \tau _i, \, x_p=0 \right\} . \end{aligned}$$

Since the *p*-th coordinate of $\sum _{i=1}^p y_i U^{(i)}$ is 0, we find that

$$\begin{aligned} P \left[ \sum _{i=1}^p y_i U^{(i)} \right] = \sum _{i =1}^{p-1} \widetilde{ y_i} \widetilde{U}^{(i)}, \end{aligned}$$

where

$$\begin{aligned} \widetilde{U}^{(i)}&= P \left[ U^{(i)} - \frac{U^{(i)}_p}{U^{(i_0)}_p} U^{(i_0)} \right] , \quad \widetilde{ y_i} = y_{i} \quad (i<i_0), \\ \widetilde{U}^{(i)}&= P \left[ U^{(i+1)} - \frac{U^{(i+1)}_p}{U^{(i_0)}_p} U^{(i_0)} \right] , \quad \widetilde{ y_i} = y_{i+1} \quad (i\ge i_0), \end{aligned}$$

and our choice of $i_0$ above ensures that $| \widetilde{U}^{(i)} |\lesssim _\epsilon 1$. Therefore,

$$\begin{aligned} \{ P x : x \in S(M,R), \, x_p=0 \} \subset \left\{ x:x= \sum _{i=1}^{p-1} \widetilde{y_i} \widetilde{U}^{(i)}, \, |\widetilde{y_i}| \lesssim \tau _{i+1} \right\} . \end{aligned}$$

We now add the condition $|x_i| \lesssim \mu _i^2$ to obtain

$$\begin{aligned}&\{ Px : x\in S(M,R), \, |x_i| \lesssim \mu _i^2 \, \,\text {if}\ 1 \le i \le p-1, \, x_p =0\} \\&\qquad \subset \left\{ x: x= \sum _{i=1}^{p-1} \widetilde{y_i} \widetilde{U}^{(i)}, \, |\widetilde{y_i}| \lesssim \tau _{i+1},\, |x_i| \lesssim \mu _i^2 \right\} \\&\qquad \subset \left\{ \sum _{t=1}^{p-1} t_i w^{(i)}: |t_i| \le 1 \right\} \qquad \text {with} \;\;\; |w^{(i)}_j| \lesssim \min (\tau _{i+1}, \mu _j^2), \end{aligned}$$

where the last inclusion is a consequence of Lemma 5.5. Applying the matrix ${\mathcal {M}}$, we see that

$$\begin{aligned}&\{ {\mathcal {M}}x:x\in S(M,R), \, |x_i| \lesssim \mu _i^2 \,\, \text {if}\ 1 \le i \le p-1, \, x_p =0\} \\&\qquad \qquad \subset \left\{ \sum _{t=1}^{p-1} t_i \widetilde{w}^{(i)} : |t_i| \le 1 \right\} \qquad \text {with} \;\;\; |\widetilde{w}^{(i)}_j| = | [{\mathcal {M}} w^{(i)}]_j |\lesssim \mu _j^{-1} \min (\tau _{i+1}, \mu _j^2). \end{aligned}$$

Finally, Lemma 5.4 gives the desired conclusion. $\square$

## Proof of Theorem 1.3

In this section we will prove the following result.

### Theorem 6.1

The following holds for any fixed off-diagonal coefficients $\beta _{ij}=\beta _{ji}\in [-\frac{1}{10d^2},\frac{1}{10d^2}]$ $(i<j)$ and almost all $(\beta _{11},\dotsc ,\beta _{dd})^T \in [1,2]^d$. Moreover it also holds for almost all symmetric matrices with $\beta _{ji}\in [-\frac{1}{10d^2},\frac{1}{10d^2}]$ for $i\ne j$ and $\beta _{11},\dotsc ,\beta _{dd}\in [1,2]$.

For every $b\in {\mathbb {N}}$, every $\delta<1<\lambda$ and any $\epsilon >0$, we have

6.1 $$\begin{aligned} \Vert P_{\lambda ,\delta } \Vert _{L^1\rightarrow L^\infty } \lesssim _{\beta ,\epsilon } \delta ^{-1/b} \lambda ^{\epsilon } \left[ \max _{0 \le b_2 \le \min (b,d+1)} \delta ^{ b_2} \lambda ^{-b_2^2 + (b+d) b_2 + {1-b}} \right] ^{1/b}. \end{aligned}$$

It follows in particular that, for every $a\in {\mathbb {N}}$ and whenever $\lambda ^{-a}\le \delta \le \lambda ^{1-a}$, we have

6.2 $$\begin{aligned} \Vert P_{\lambda ,\delta } \Vert _{L^1\rightarrow L^\infty } \lesssim _{\beta ,\epsilon } {\left\{ \begin{array}{ll} \delta ^{1- \frac{1}{d+1-a}} \lambda ^{d - 1 + {\frac{1}{d+1-a}} + \epsilon } &{}(a<d), \\ {\delta ^{1-\frac{1+a}{d+1+a}} \lambda ^{ d -1+\frac{1+a}{d+1+a}+ \epsilon }} &{}( a\ge d). \end{array}\right. } \end{aligned}$$

We emphasize that (6.2) is intended as illustrative; one could prove a stronger but less tidy result just by making a more careful choice of the parameter *b* at the end of the proof.

By contrast it seems more challenging to improve (6.1) using our methods. See Remark 6.2 for one idea.

Before proceeding to the proof we deduce Theorem 1.3.

### Proof of Theorem 1.3

Equations (1.10) and (1.9) are cases of (6.2). For (1.8), observe that if $\Vert P_{\lambda ,\delta }\Vert$ is sufficiently large in terms of the function $\chi$, then there are two integer vectors with

$$\begin{aligned} {{\mathcal {Q}}(x^{(1)})}^{1/2}, {{\mathcal {Q}}(x^{(2) })}^{1/2} \in [\lambda -\delta ,\lambda +\delta ], \quad \text {and}\quad |x^{(1)}_i|\ne |x^{(2)}_i| \quad \text { for some}\ i. \end{aligned}$$

Letting $y_{ij}=x^{(1)}_ix^{(1)}_j-x^{(2)}_ix^{(2)}_j$ we find that

6.3 $$\begin{aligned} y_{ij} \in {\mathbb {Z}}, \quad |y_{ij}| \lesssim \lambda ^2, \quad y_{ii} \ne 0\text { for some }i, \quad \Big |\sum _{i.j} \beta _{ij}y_{ij} \Big |\lesssim \delta \lambda . \end{aligned}$$

For a given matrix $y_{ij}$ and for any off-diagonal coefficients $\beta _{ij}=\beta _{ji}$ $(i<j)$ we have

$$\begin{aligned} \textrm{mes}\Big \{(\beta _{11},\dotsc ,\beta _{dd})^T\in [1,2]^d : \Big |\sum _{i.j} \beta _{ij}y_{ij} \Big |\le \delta \lambda \Big \} \lesssim \frac{\delta \lambda }{\max \{|y_{ij}|\}}. \end{aligned}$$

We claim that

$$\begin{aligned} \#\{(y_{ij}) : y_{ij}=x^{(1)}_ix^{(1)}_j-x^{(2)}_ix^{(2)}_j\text { for some }x^{(i)}\in {\mathbb {Z}}^d,\text { and } 0<\max \{|y_{ij}|\}\le Y \} \lesssim _\epsilon Y^{d+\epsilon }. \end{aligned}$$

For the proof, note that we cannot have every $$y_{ii}=0$$ or else $$(y_{ij})$$ would vanish. We assume without loss of generality that there is $$i_0\in \{1,\dotsc ,d\}$$ such that $$y_{ii}=0$$ iff $$i> i_0$$. There are $$O(Y^{i_0})$$ possible values of $$y_{ii}$$ for $$i\le i_0$$, and once these are chosen the identity $$y_{ii} =(x^{(1)}_i+x^{(2)}_i)(x^{(1)}_i-x^{(2)}_i) $$ determines $$x^{(1)}_i,x^{(2)}_i$$ for $$i\le i_0$$ up to $$Y^\epsilon $$ possibilities. Next for $$i>i_0$$ we have $$x^{(1)}_i=\pm x^{(2)}_i$$, and so up to finitely many choices both of these are determined by the values of $$y_{1i} = (\pm x^{(1)}_1\pm x^{(2)}_1)x^{(1)}_i$$, for which there are $$O(Y^{d-i_0})$$ possibilities. We conclude that there are $$\lesssim _\epsilon Y^{d+\epsilon }$$ choices for the $$x^{(i)}$$ and hence for $$(y_{ij})$$.

We can now conclude that, for a suitably large constant *C* depending only on the cutoff function $\chi$, and for any fixed values of the off-diagonal entries $|\beta _{ij}|\le \frac{1}{10d^2}$ ($i< j$), we have

$$\begin{aligned}&\textrm{mes}\{(\beta _{11},\dotsc ,\beta _{dd})^T\in [1,2]^d : \Vert P_{\lambda ,\delta }\Vert >C\text { for some } \lambda \sim \lambda _0, \delta< \delta _0\}\\&\quad \lesssim \sum _{\begin{array}{c} (y_{ij})\\ 0<\max \{|y_{ij}|\}\lesssim \lambda _0^2 \end{array}} \frac{\delta _0\lambda _0}{\max \{|y_{ij}|\} } \\&\quad \lesssim _\epsilon \sum _{\begin{array}{c} Y=2^k \\ k\in {\mathbb {Z}}\\ Y\lesssim \lambda _0^2 \end{array}} \delta _0\lambda _0Y^{d-1+\epsilon }. \end{aligned}$$

This is $O_\epsilon (\delta _0\lambda _0^{2d-1})$. Applying the Borel-Cantelli lemma (Lemma A.1) proves (1.8). $\square$

We now begin the proof of Theorem 6.1. Throughout the rest of this section we write $\beta _i$ for $\beta _{ii}$, put $\beta '=(\beta _1,\dotsc \beta _d)^T$, and given $b,d\in {\mathbb {N}}$ and $M = (m_{ij})_{1\le i\le d,\,1\le j\le b}$, we put

$$\begin{aligned} P(M)=(p_{ij}(M))_{1\le i \le d+1,\,1\le j\le b} =\left( \begin{matrix} m^2_{11}&{}\cdots &{}m^2_{1b}\\ \vdots &{}\ddots &{}\vdots \\ m^2_{d1}&{}\cdots &{}m^2_{db}\\ \lambda _0^2&{}\cdots &{}\lambda _0^2 \end{matrix}\right) . \end{aligned}$$

### Integrating over $\lambda$ and $\beta$

Our key observation is as follows. Since, for $m\in {\mathbb {Z}}$, we have $1\le \sum _{\mu \in 2^{{\mathbb {N}}}\cup \{0\}} \textbf{1}_{\mu \le 2m\le 2\mu },$ and since $\chi$ takes non-negative values, we have for any $\lambda ,\delta >0$ that

$$\begin{aligned}&\int _{[1,2]^d} \int _{\lambda _0/2}^{\lambda _0} \Vert P_{\lambda ,\delta } \Vert _{L^1\rightarrow L^\infty }^b \, d \lambda \, d {\beta '} \\&\quad = \int _{[1,2]^d} \int _{\lambda _0/2}^{\lambda _0} \bigg (\sum _{\begin{array}{c} \lambda _0 \ge \mu _1,\dotsc , \mu _d \ge 0\\ \mu _i \in 2^{{\mathbb {N}}}\cup \{0\} \end{array}} \sum _{\begin{array}{c} {m} \in {\mathbb {Z}}^d \\ |m_i|\in [\frac{\mu _i}{2},\mu _i] \end{array}} \chi \left( \frac{{\mathcal {Q}}({m}) - \lambda ^2}{\delta \lambda } \right) \bigg )^b\, d \lambda \, d {\beta '}\\&\quad \lesssim \int _{[1,2]^d} \int _{\lambda _0/2}^{\lambda _0} \log ^{bd}(\lambda _0) \max _{\begin{array}{c} \lambda _0 \ge \mu _1\ge \cdots \ge \mu _d \ge 0\\ \mu _i \in 2^{{\mathbb {N}}}\cup \{0\} \end{array}} \bigg (\sum _{\begin{array}{c} {m} \in {\mathbb {Z}}^d \\ |m_i|\in [\frac{\mu _i}{2},\mu _i] \end{array}} \chi \left( \frac{{\mathcal {Q}}({m}) - \lambda ^2}{\delta \lambda } \right) \bigg )^b\, d \lambda \, d {\beta '}\\&\quad {\lesssim \int _{[1,2]^d} \int _{\lambda _0/2}^{\lambda _0} \log ^{bd}(\lambda _0) \max _{\begin{array}{c} \lambda _0 \ge \mu _1\ge \cdots \ge \mu _d \ge 0\\ \mu _i \in 2^{{\mathbb {N}}}\cup \{0\} \end{array}} \prod _{j=1}^b \sum _{\begin{array}{c} m_{1j},\dotsc ,m_{dj}\in {\mathbb {Z}} \\ |m_{ij}| \in \left[ \frac{\mu _i}{2},\mu _i\right] \end{array}} \textbf{1}_{ |{{\mathcal {Q}}(m_{1j},\dotsc ,m_{dj}) - \lambda ^2}|\le \delta \lambda } \, d \lambda \, d {\beta '},} \end{aligned}$$

and if we temporarily write the off-diagonal parts of ${\mathcal {Q}}(m_{1j},\dotsc ,m_{dj})$ using the row vector

$$\begin{aligned} q=\Big (\sum _{\begin{array}{c} 1\le i,j\le d \\ i \ne j \end{array}} \beta _{ij} m_{i1}m_{j1},\dotsc , \sum _{\begin{array}{c} 1\le i,j\le d \\ i \ne j \end{array}} \beta _{ij} m_{ib}m_{jb}\Big ). \end{aligned}$$

then this becomes

6.4 $$\begin{aligned}&\int _{[1,2]^d} \int _{\lambda _0/2}^{\lambda _0} \Vert P_{\lambda ,\delta } \Vert _{L^1\rightarrow L^\infty }^b \, d \lambda \, d {\beta '}\nonumber \\&\quad \lesssim \int _{[1,2]^d} \int _{\lambda _0/2}^{\lambda _0} \log ^{bd}(\lambda _0) \max _{\begin{array}{c} \lambda _0 \ge \mu _1\ge \cdots \ge \mu _d \ge 0\\ \mu _i \in 2^{{\mathbb {N}}}\cup \{0\} \end{array}} \prod _{j=1}^b \sum _{\begin{array}{c} m_{1j},\dotsc ,m_{dj}\in {\mathbb {Z}} \\ |m_{ij}|\in \left[ \frac{\mu _i}{2},\mu _i\right] \end{array}} \textbf{1}_{ |(\beta _1,\dotsc ,\beta _d, - \lambda ^2/\lambda _0^2) P(M)-q|\le \delta \lambda _0}\nonumber \\&\quad d \lambda \, d {\beta '}= \log ^{bd}(\lambda _0) \max _{\begin{array}{c} \lambda _0 \ge \mu _1\ge \cdots \ge \mu _d \ge 0\\ \mu _i \in 2^{{\mathbb {N}}}\cup \{0\} \end{array}} \nonumber \\&\quad \sum _{\begin{array}{c} M\in {\mathbb {Z}}^{d\times b} \\ |m_{ij}| \in [\mu _i/2,\mu _i] \end{array}}\textrm{mes}\big \{({\beta '},\lambda )\in [1,2]^d\times \left[ \frac{\lambda _0}{2},\lambda _0\right] : |(\beta _1,\dotsc ,\beta _d, - \lambda ^2/\lambda _0^2) P(M)-q|\le \delta \lambda _0\big \}, \end{aligned}$$

We can estimate the measure inside the last sum in (6.4) as follows. Notice first that

6.5 $$\begin{aligned} D_1(P(M)) = \max _{\begin{array}{c} i=1,\dotsc , d+1 \\ j=1,\dotsc ,b \end{array}} |p_{ij}(M) |\sim \lambda _0^2. \end{aligned}$$

By Lemma 5.1, we have

$$\begin{aligned}&\textrm{mes}\Big \{ {\gamma }\in {\mathbb {R}}^{d+1} : |{\gamma }|\le 1, \, |\sum _i \gamma _ip_{ik}(M) - \sum _{i \ne j} \beta _{ij} m_{ik}m_{jk} |\le \delta \lambda _0 \Big \} \\&\quad \lesssim \prod _{k=1}^{\min (b,d+1)} \min \left( 1, \frac{\delta \lambda _0}{\sigma _k(P(M))} \right) = \prod _{\begin{array}{c} 1 \le i \le \min (b,d+1) \\ \sigma _i(P(M))>\delta \lambda _0 \end{array}} \frac{\delta \lambda _0}{\sigma _i(P(M))}. \end{aligned}$$

Together with (6.4) and the fact that *P*(*M*) does not depend on the signs of the $m_{ij}$, we find

$$\begin{aligned}&\int _{[1,2]^d} \int _{\lambda _0/2}^{\lambda _0} \Vert P_{\lambda ,\delta } \Vert _{L^1\rightarrow L^\infty }^b \, d \lambda \, d {\beta '} \\&\quad \lesssim (\log \lambda _0)^{bd+d} \max _{\begin{array}{c} \lambda _0 \ge \mu _1\ge \cdots \ge \mu _d \ge 0\\ \mu _i \in 2^{{\mathbb {N}}}\cup \{0\} \end{array}} \sum _{\begin{array}{c} M\in {\mathbb {Z}}^{d\times b} \\ m_{ij} \in [\mu _i/2,\mu _i] \end{array}} \lambda _0 \prod _{\begin{array}{c} 1 \le i \le \min (b,d+1) \\ \sigma _i(P(M))>\delta \lambda _0 \end{array}} \frac{\delta \lambda _0}{\sigma _i(P(M))}, \end{aligned}$$

where the $m_{ij}$ are now non-negative since $m_{ij} \in [\mu _i/2,\mu _i]$. Combining this with (6.5) and the Corollary to Lemma 5.2 yields

6.6 $$\begin{aligned}&\int _{[1,2]^d} \int _{\lambda _0/2}^{\lambda _0} \Vert P_{\lambda ,\delta } \Vert _{L^1\rightarrow L^\infty }^b \, d \lambda \, d {\beta '} \nonumber \\&\quad \lesssim (\log \lambda _0)^{bd+d} \max _{\begin{array}{c} \lambda _0^2 \sim L_1\ge \cdots \ge L_{{\min (b,d+1)}} \ge 0 \\ \lambda _0 \ge \mu _1\ge \cdots \ge \mu _d \ge 0 \\ \mu _i \in 2^{{\mathbb {N}}}\cup \{0\} \end{array}} \lambda _0 \prod _{\begin{array}{c} 1 \le i \le \min (b,d+1) \\ L_i>\delta \lambda _0 \end{array}} \frac{\delta \lambda _0}{L_i} Z_{d,b}(\vec {\mu },\vec {L}), \end{aligned}$$

where

6.7 $$\begin{aligned} Z_{d,b}(\vec {\mu },\vec {L})&= \#\Big \{ M\in {\mathbb {Z}}^{d\times b} : \frac{m_{ij}}{\mu _i }\in [\tfrac{1}{2},1],\, \frac{ D_k(P(M))}{L_1\cdots L_k }\in [\tfrac{1}{2},1]\nonumber \\&\qquad \quad \text {for all } 1\le i \le d,\;1\le j\le b,\;1 \le k \le {\min (b,d+1)} \Big \}. \end{aligned}$$

In (6.6) we may assume that $(\mu _i=0\implies L_{i+1}=0)$, since otherwise $Z_{d,b}(\vec {\mu },\vec {L})$ would be zero (there are no such *M*). In particular allowing $\mu _i$ to be zero is the same as allowing the dimension *d* to drop, in the sense that

$$\begin{aligned}&\max _{\begin{array}{c} \lambda _0^2 \sim L_1\ge \cdots \ge L_{{\min (b,d+1)}} \ge 0 \\ \lambda _0 \ge \mu _1\ge \cdots \ge \mu _d \ge 0\\ \mu _i \in 2^{{\mathbb {N}}}\cup \{0\} \end{array}} \lambda _0 \left[ \prod _{\begin{array}{c} 1 \le i \le \min (b,d+1) \\ L_i>\delta \lambda _0 \end{array}} \frac{\delta \lambda _0}{L_i} \right] Z_{d,b}(\vec {\mu },\vec {L})\\&\quad = \max _{\begin{array}{c} 0\le d'\le d\\ \lambda _0^2 \sim L_1\ge \cdots \ge L_{{\min (b,d'+1)}} \ge 0 \\ \lambda _0 \ge \mu _1\ge \cdots \ge \mu _{d'} \ge 1,\,\mu _i \in 2^{{\mathbb {N}}} \end{array}}\lambda _0\left[ \prod _{\begin{array}{c} 1 \le i \le \min (b,d'+1) \\ L_i>\delta \lambda _0 \end{array}} \frac{\delta \lambda _0}{L_i} \right] Z_{d',b}(\vec {\mu },\vec {L}). \end{aligned}$$

### Counting matrices with prescribed subdeterminants

We want to estimate the right-hand side of (6.6), under the assumption that $\underline{\mu _i {\ne } 0\ \text {for any}\ i}$, or in other words $\mu _i \in 2^{\mathbb {N}}$.

Our first object is to estimate from above the number of matrices *M* counted by the function $Z_{d,b}(\vec {\mu },\vec {L})$ from (6.7). By Lemma 5.3, it suffices to l count those *M* satisfying an additional condition

$$\begin{aligned} D_k(P(M))\sim D_k^{(k)}(P(M)) {\text { for all }} \le k \le {\min (b,d+1)}, \end{aligned}$$

since permuting the columns of these recovers all the matrices in $Z_{d,b}(\vec {\mu },\vec {L})$

For $j=1,\dotsc ,b$ define the vectors $m^{(j)}$ and ${n}^{(j)}\in {\mathbb {R}}^d$ by

$$\begin{aligned} m^{(j)} = {(m_{1j},\dotsc ,m_{dj})^T,} \qquad {n}^{(j)} = (m_{1j}^2/\mu _1,\dotsc ,m_{dj}^2/\mu _d)^T. \end{aligned}$$

That is the vectors $m^{(j)}$ are the columns of *M*. Meanwhile the vectors $n^{(j)}$ are the columns of *P*(*M*) with the last element dropped and the others rescaled to that $n^{(j)}$ belongs to the set *S* defined by

$$\begin{aligned} S = \left\{ (u_{1}^2/\mu _1,\dotsc ,u_{d}^2/\mu _d)^T: u_i\in {\mathbb {Z}},\, u_i \in \left[ \frac{\mu _i}{2},\mu _i\right] \right\} , \end{aligned}$$

whose elements are separated by gaps of size $\sim 1$. If *M* is counted in the right-hand side of (6.7), then the vector ${n}^{(1)}$ can be chosen arbitrarily from *S*; there are ${\lesssim } \prod _{i=1}^d \mu _i$ choices.

Suppose now that the first *k* columns of *P*(*M*) are given, and they satisfy

$$\begin{aligned} D_\ell (P(m^{(1)}|\cdots | m^{(k)})) \sim L_1\cdots L_\ell \qquad (1\le \ell \le \min ( k,d+1)). \end{aligned}$$

We want to select $m^{(k+1)}$, or equivalently $n^{(k+1)}$. We can first use that *S* is 1-separated to replace our counting problem by a volume estimate: letting

$$\begin{aligned} {\mathcal {N}}^{k+1}&= \{ n^{(k+1)} \in S: {m^{(k)}_i \in [\mu _i/2,\mu _i],} \, D_\ell (P(m^{(1)},\dots m^{(k+1)})) \sim L_1 \dots L_\ell \\&\qquad \quad \,\,(1\le i \le d, \,1\le \ell \le \min (k+1,d+1)) \}, \end{aligned}$$

then

$$\begin{aligned} \# {\mathcal {N}}^{k+1} \lesssim \textrm{mes}\left[ {\mathcal {N}}^{k+1} + B(0,1) \right] . \end{aligned}$$

#### Remark 6.2

This volume bound is not necessarily optimal. To take just one simple example, if $\lambda _0^2$ is an integer then $D_\ell ^{(\ell )}(P(M))$ is an integer. Thus, in the case when $0<L_1\cdots L_\ell \ll 1$, it is impossible for $D_\ell ^{(\ell )}(P(M))\sim L_1\cdots L_\ell$ to hold and the set ${\mathcal {N}}^{k+1}$ is empty.

We apply Lemma 5.7 with

$$\begin{aligned} p&=d+1, \quad M ={P(m^{(1)}|\dots |m^{(k)}),} \quad {\mathcal {M}}x&=n^{(k+1)}, \quad A =\lambda _0^2, \quad R=L_1\cdots L_{k+1}. \end{aligned}$$

We compute that

$$\begin{aligned} \tau _i \asymp {\left\{ \begin{array}{ll} \max \{L_i,L_{k+1}\} &{}(k\le d), \\ L_i &{}(k>d). \end{array}\right. } \end{aligned}$$

We now need to distinguish two cases. If $k\le d$ then applying Lemma 5.7 gives that

$$\begin{aligned} \# {\mathcal {N}}^{k+1} \lesssim 1+ \max _{\begin{array}{c} {\mathcal {I}}\subset \{1,\dotsc ,d\} \\ {\mathcal {J}}\subset \{1,\dotsc ,d\} \\ \#{\mathcal {I}}=\#{\mathcal {J}}\\ \sigma : {\mathcal {I}}\hookrightarrow {\mathcal {J}} \end{array}} \bigg (\prod _{i\in {\mathcal {I}}} \mu _i \bigg ) \bigg ( \prod _{\begin{array}{c} \mu _i^2 > \max (L_{\sigma (i)+1},L_{k+1})\\ i\in {\mathcal {I}} \end{array}} \frac{\max (L_{\sigma (i)+1},L_{k+1})}{\mu _i^2} \bigg ). \end{aligned}$$

If instead $k >d$, applying Lemma 5.7 gives that

$$\begin{aligned} \# {\mathcal {N}}^{k+1} \lesssim 1+ \max _{\begin{array}{c} {\mathcal {I}}\subset \{1,\dotsc , d\} \\ {\mathcal {J}}\subset \{1,\dotsc ,d\} \\ \#{\mathcal {I}}=\#{\mathcal {J}}\\ \sigma : {\mathcal {I}}\hookrightarrow {\mathcal {J}} \end{array}} \bigg (\prod _{i\in {\mathcal {I}}} \mu _i \bigg ) \bigg ( \prod _{\begin{array}{c} {\mu _i^2 > L_{\sigma (i)+1}}\\ i\in {\mathcal {I}} \end{array}} \frac{ L_{\sigma (i)+1}}{\mu _i^2} \bigg ). \end{aligned}$$

Recall now that this is a bound for the number of choices for $m^{(k+1)}$, given $m^{(1)},\dotsc ,m^{(k)}$, and that there are $\mu _1\cdots \mu _d$ choices for $m^{(1)}$. Recall also that our object is to estimate that part of the right-hand side of (6.6) for which every $\mu _i$ is nonzero. The bound we have proved is

6.8 $$\begin{aligned}&\max _{\begin{array}{c} \lambda _0^2 \sim L_1\ge \cdots \ge L_{{\min (b,d+1)}} \ge 0 \\ \lambda _0 \ge \mu _1\ge \cdots \ge \mu _d> 0 \\ \mu _i \in 2^{{\mathbb {N}}} \end{array}} \lambda _0 \prod _{\begin{array}{c} 1 \le i \le \min (b,d+1) \\ L_i>\delta \lambda _0 \end{array}} \frac{\delta \lambda _0}{L_i} Z_{d,b}(\vec {\mu },\vec {L}) \nonumber \\&\quad \lesssim \max _{ \begin{array}{c} \lambda _0^2 \sim L_1\ge \cdots \ge L_{\min (b,d+1)} \ge 0 \\ \lambda _0 \ge \mu _1\ge \cdots \ge \mu _d \ge 0 \end{array}} \max _{\begin{array}{c} {\mathcal {I}}_k \subset \{1,\dotsc ,d\} \\ \sigma _k: {\mathcal {I}}_k \hookrightarrow \{1,\dots , d \} \end{array}} {\mathcal {F}}((L_i),(\mu _i),({\mathcal {I}}_k),(\sigma _k)), \end{aligned}$$

where $\sigma _k$ is a bijection from ${\mathcal {I}}_k$ to a subset of $\{1,\dots ,d\}$ and

$$\begin{aligned}&{\mathcal {F}}((L_i),(\mu _i),({\mathcal {I}}_k),(\sigma _k)) \\&\quad = \lambda _0 \bigg (\prod _{\begin{array}{c} i=1,\dotsc ,\min (b,d+1) \\ L_i>\delta \lambda _0 \end{array}} \frac{\delta \lambda _0}{L_i}\bigg ) \bigg (\prod _{i=1}^d \mu _i \bigg ) \prod _{k=1}^{\min (b-1,d)} \bigg (\prod _{i\in {\mathcal {I}}_k} \mu _i \bigg )\\&\qquad \bigg ( \prod _{\begin{array}{c} \mu _i^2> \max \{L_{\sigma _k(i)+1},L_{k+1}\}\\ i\in {\mathcal {I}}_k \end{array}} \frac{\max (L_{\sigma _k(i)+1},L_{k+1}) }{\mu _i^2} \bigg ) \\&\qquad \prod _{k=\min (b,d+1)}^{b-1} \bigg (\prod _{i\in {\mathcal {I}}_k} \mu _i \bigg ) \bigg ( \prod _{\begin{array}{c} \mu _i^2 >L_{\sigma _k(i)+1}\\ i\in {\mathcal {I}}_k \end{array}} \frac{L_{\sigma _k(i)+1}}{\mu _i^2} \bigg ), \end{aligned}$$

with the understanding that ${\mathcal {I}}$ might be empty and $\prod _{{\mathcal {I}}} = 1$ if ${\mathcal {I}} = \emptyset$.

### The maximization procedure

Our aim is now to find the values of $L_i,\mu _i,{\mathcal {I}}_k,\sigma _k$ for which the maximum on the right-hand side of (6.8) is attained.

$\underline{\text {Step 1: Maximizing in} \ (L_i)\ \text {with the}\ {\mathcal {I}}_k'\text {s and} \ \mu _i'\text {s held fixed.}}$ We start with the dependence on $(L_i)$, and we relax first the condition that they be ordered; we will simply assume that $0 \le L_i \le \lambda _0^2$ for each $i=1, \dotsc , \min (b,d+1)$. Next, we claim that the products

$$\begin{aligned} \bigg ( \prod _{\begin{array}{c} \mu _i^2> \max (L_{\sigma _k(i)+1},L_{k+1}) \\ i\in {\mathcal {I}}_k \end{array}} \frac{\max (L_{\sigma _k(i)+1},L_{k+1}) }{\mu _i^2} \bigg ) \quad \text {and} \quad \bigg ( \prod _{\begin{array}{c} \mu _i^2 >L_{\sigma _k(i)+1}\\ i\in {\mathcal {I}}_k \end{array}} \frac{L_{\sigma _k(i)+1}}{\mu _i^2} \bigg ) \end{aligned}$$

on the right-hand side of the definition of ${\mathcal {F}}((L_i),(\mu _i),({\mathcal {I}}_k),(\sigma _k))$ can be taken to be empty. Indeed, assume that the maximum of ${\mathcal {F}}$ is reached at a point such that, for some *k* in the product, the corresponding product is not empty: it contains $\frac{\max (L_{\sigma _k(i)+1},L_{k+1})}{\mu _i^2}$ or $\frac{L_{\sigma _k(i)+1}}{\mu _i^2}$ for some *i*; we will denote it $\frac{L_{i_0}}{\mu _{i_1}^2}$. Expand ${\mathcal {F}}$ in powers of the $L_i$, and consider the exponent of $L_{i_0}$. It is necessarily $\ge 0$, since only the first factor in the definition of ${\mathcal {F}}$ contributes a negative power, namely $L_{i_0}^{-1}$. Therefore, ${\mathcal {F}}$ will be larger, or equal, if we increase the value of $\max (L_{\sigma _k(i)+1},L_{k})$ to $\mu _{i_1}^2$, which has the effect of cancelling the undesirable term.

After this manipulation, the parentheses we mentioned have been cancelled, and the value of some of the $L_i$’s has been fixed to $\mu _{f(i)}^2$ for some function *f*. The remaining $L_i$ contribute $\textbf{1}_{L_i > \delta \lambda _0} \frac{\delta \lambda _0}{L_i }$, and they might be constrained by inequalities of the type $L_i > \mu _j^2$. Therefore, ${\mathcal {F}}$ will be maximal if they take the value $\delta \lambda _0$, or $\mu _{f(i)}^2$ for some function *f*.

$\underline{\text {Step 2: Maximizing in}\ (\mu _i).}$ The result of the maximization in $(L_i)$ is that we can assume that each $L_i$ takes the value either $\mu _{f(i)}^2$, or $\delta \lambda _0$, that $\mu _i^2 \le \max (L_{\sigma _k(i)+1},L_{k+1}) \; \text {if}\ i \in {\mathcal {I}}_k$, with the convention that $L_{k} = 0$ for $k \ge d+2$, and that the function to maximize is

6.9 $$\begin{aligned} \lambda _0\bigg (\prod _{\begin{array}{c} i=1,\dotsc ,\min (b,d+1) \\ L_i>\delta \lambda _0 \end{array}} \frac{\delta \lambda _0}{L_i}\bigg ) \bigg (\prod _{i=1}^d \mu _i \bigg ) \prod _{k=1}^{b-1} \bigg (\prod _{i\in {\mathcal {I}}_k} \mu _i \bigg ). \end{aligned}$$

We now claim that, at the maximum, the $(\mu _i)$ either take the value 1 or $\lambda _0$. To prove this claim, assume that, at the maximum, the $\mu _i$ take a number *n* of distinct values $1 \le a_1< \dots < a_n \le \lambda _0$. Replacing $L_i$ by $\mu _{f(i)}^2$ in the above expression, it takes the form

$$\begin{aligned} \lambda _0 (\lambda _0 \delta )^{\alpha _0} \prod _{i=1}^n a_i^{\alpha _i}, \qquad \text {where}\ \alpha _i \in {\mathbb {Z}}. \end{aligned}$$

If $\alpha _i >0$ and $a_i < \lambda _0$, then this expression will increase if the value of $a_i$ is increased until $a_{i+1}$ or $\lambda _0$; and similarly, if $\alpha _i <0$ and $a_i > 1$, it will decrease if the value of $a_i$ is decreased until $a_{i-1}$ or 1. This contradicts the maximality of $(\mu _i)$ unless $a_i$ only takes the values $\lambda _0$ or 1 for $\alpha _i \ne 0$. There remains the case where $\alpha _i =0$, but then $\mu _i$ can be assigned the value $\lambda _0$ or 1 indifferently.

$\underline{\text {Step 3: Maximizing in} \ ({\mathcal {I}}_k)\ \text {and}\ (\sigma _k).}$ We showed that the maximum of ${\mathcal {F}}$ is less than the maximum of (6.9), under the constraint that $\mu _i^2 \le \max (L_{\sigma _k(i)+1},L_{k+1})$ if $i \in {\mathcal {I}}_k$ (with the convention that $L_{k} = 0$ for ${k \ge d+2}$); and under the further constraint that $L_i$ can only take the values $\delta \lambda _0,1,\lambda _0$, and $\mu _i$ can only take the values $1,\lambda _0$.

There are now two cases to consider:

- If $k\le \min (b-1,d)$ and $L_{k+1} = \lambda _0^2$, then the optimal choice for ${\mathcal {I}}_k$ is $\{ 1,\dots ,d \}$.
- Otherwise, ${\mathcal {I}}_k$ should have the same cardinal as the set of $L_i$, $i \ge 2$, equal to $\lambda _0^2$, and $\sigma _k +1$ should map ${\mathcal {I}}_k$ to this set.

Step 4: Conclusion. As a result of the previous reductions, we find

$$\begin{aligned}&\max _{ \begin{array}{c} \lambda _0^2 \sim L_1\ge \cdots \ge L_{\min (b,d+1)} \ge 0 \\ \lambda _0 \ge \mu _1\ge \cdots \ge \mu _d \ge 0 \end{array}} \max _{\begin{array}{c} {\mathcal {I}}_k \subset \{1,\dotsc ,d\} \\ \sigma _k: {\mathcal {I}}_k \hookrightarrow \{1,\dots , d \} \end{array}} {\mathcal {F}}((L_i),(\mu _i),({\mathcal {I}}_k),(\sigma _k)) \\&\quad \lesssim \max _{\begin{array}{c} \lambda _0=L_1\ge \cdots \ge L_{\min (b,d+1)} \\ \mu _1\ge \cdots \ge \mu _d \\ \\ L_i \in \{\delta \lambda _0,1,\lambda _0^2\}, \mu _i\in \{1,\lambda _0\} \end{array}} \lambda _0 \bigg ( \prod _{\begin{array}{c} i=1,\dotsc ,\min (b,d+1) \\ L_i>\delta \lambda _0 \end{array}} \frac{\delta \lambda _0}{L_i}\bigg ) \bigg (\prod _{i=1}^d \mu _i \bigg ) \prod _{k=1}^{b-1} \bigg ({\prod _{\begin{array}{c} i\in \{1,\dotsc ,d\} \\ L_{i+1}=\lambda _0^2,\text { or} \\ (k\le d \text { and }L_{k+1}=\lambda _0^2) \end{array}}} \mu _i \bigg ). \end{aligned}$$

Notice that we are assuming again that the $(\mu _i)$ and $(L_i)$ are ordered; a moment of reflection shows that this is possible since the permutation $(\sigma _k)$ can be freely chosen. This expression is visibly nondecreasing in $(\mu _i)$, so we might as well take all $\mu _i$ to be $\lambda _0$.

In order to evaluate the resulting expression, we need to know the number of $L_k$ equal to, respectively, $\delta \lambda _0,1,\lambda _0$; this is also the information needed to determine ${\mathcal {I}}_k$; therefore, we define the numbers

$$\begin{aligned} b_0&= \#\{1\le j\le \min (b,d+1) : L_j = \delta \lambda _0\}, \\ b_1&= \#\{1\le j\le \min (b,d+1) : L_j = 1\}, \\ b_2&= \#\{1\le j\le \min (b,d+1) : L_j = \lambda _0^2\}, \end{aligned}$$

which are such that

$$\begin{aligned} b_0 + b_1 + b_2 = \min (b,d+1). \end{aligned}$$

Letting $\chi =1$ if ${1>\delta \lambda _0}$ and $\chi =0$ otherwise, the maximization procedure shows that the function to be optimized is bounded by

$$\begin{aligned}&\max _{b_0+b_1+b_2=\min (b,d+1)} \lambda _0 (\delta \lambda _0)^{b_1\chi + b_2} \lambda _0^{-2 b_2} \lambda _0^d \lambda _0^{(b_2-1) d} \lambda _0^{(b - b_2)(b_2-1)} \\&\quad = \max _{b_1+b_2\le \min (b,d+1)} {(\delta \lambda _0)^{b_1\chi }\delta ^{b_2}} \lambda _0^{-b_2^2 + (b+d) b_2 + 1-b} \end{aligned}$$

We notice first that $b_1$ can be taken to be zero. Second, there remains to dispose of the assumption that all $\mu _i$ are non-zero, which was made at the beginning of Sect. 6.2. By the comments just prior to the start of Sect. 6.2, this equivalent to reducing the dimension *d*. But some thought shows that the above expression is increasing with *d*, so that allowing for smaller *d* is harmless. Overall, the final bound we find is

$$\begin{aligned}{} & {} \max _{ \begin{array}{c} \lambda _0^2 \sim L_1\ge \cdots \ge L_{\min (b,d+1)} \ge 0 \\ \lambda _0 \ge \mu _1\ge \cdots \ge \mu _d \ge 0 \end{array}} \max _{\begin{array}{c} {\mathcal {I}}_k \subset \{1,\dotsc ,d\} \\ \sigma _k: {\mathcal {I}}_k \hookrightarrow \{1,\dots , d \} \end{array}} {\mathcal {F}}((L_i),(\mu _i),({\mathcal {I}}_k),(\sigma _k))\\{} & {} \quad \lesssim \max _{b_2 \le \min (b,d+1)} \delta ^{ b_2} \lambda _0^{-b_2^2 + (b+d) b_2 + 1-b}. \end{aligned}$$

### Borel-Cantelli and the end of the argument

The three previous subsections give the estimate

$$\begin{aligned} \int _{[1,2]^d} \int _{\lambda _0/2}^{\lambda _0} \Vert P_{\lambda ,\delta } \Vert _{L^1\rightarrow L^\infty }^b \, d \lambda \, d {\beta '} \lesssim (\log \lambda _0)^{bd+d}\max _{b_2 \le \min (b,d+1)} \delta ^{ b_2} \lambda _0^{-b_2^2 + (b+d) b_2 + 1-b}, \end{aligned}$$

valid uniformly for any off-diagonal coefficients Since $P_{\lambda ,\delta }$ varies on a scale $\sim \delta$, this implies that, for $\lambda \in [\frac{\lambda _0}{2}, \lambda _0]$,

$$\begin{aligned} \int _{[1,2]^d} \sup _{\frac{\lambda _0}{2}< \lambda < \lambda _0} \Vert P_{\lambda ,\delta } \Vert _{L^1\rightarrow L^\infty }^b \, d \beta ' \lesssim (\log \lambda _0)^{bd+d} \delta ^{-1} \max _{b_2 \le \min (b,d+1)} \delta ^{ b_2} \lambda _0^{-b_2^2 + (b+d) b_2 + 1-b}. \end{aligned}$$

By Borel-Cantelli as in Appendix A, this implies that, for any $\epsilon > 0$,

6.10 $$\begin{aligned} \Vert P_{\lambda ,\delta } \Vert _{L^1\rightarrow L^\infty } \lesssim _{\beta ,\epsilon } \delta ^{-1/b} \lambda ^{\epsilon } \left[ \max _{b_2 \le \min (b,d+1)} \delta ^{ b_2} \lambda ^{-b_2^2 + (b+d) b_2 + 1-b} \right] ^{1/b}, \end{aligned}$$

both for fixed off-diagonal coefficients and almost all $\beta '\in [1,2]^d$, and also for almost all matrices $(\beta _{ij})$ with $\beta '\in [1,2]^d$ and small off-diagonal coefficients. This proves (6.1).

We now observe that if $b_2$ is strictly less than $\min (b,d+1)$, then

$$\begin{aligned} \frac{ \delta ^{ b_2+1} \lambda ^{-(b_2+1)^2 + (b+d) (b_2+1) +1-b} }{\delta ^{ b_2} \lambda ^{-b_2^2 + (b+d) b_2 + 1-b}}&=\delta \lambda ^{-2b_2-1+b+d} \\&\ge \delta \lambda ^{-2\min (b-1,d)-1+b+d}. \end{aligned}$$

This will be $\ge 1$ provided that $\delta \ge \lambda ^{\min (b-d-1,d+1-b)}$, and so for such $\delta$ the maximum in (6.10) is reached for $b_2 = \min (b,d+1)$. We will therefore impose the condition $\delta \ge \lambda ^{\min (b-d-1,d+1-b)}$ for convenience rather than because we believe it to be optimal. This yields

$$\begin{aligned} \Vert P_{\lambda ,\delta } \Vert _{L^1\rightarrow L^\infty } \lesssim _{\epsilon ,\beta '} {\left\{ \begin{array}{ll} \delta ^{1- \frac{1}{b}} \lambda ^{d - 1 + {\frac{1}{b}} + \epsilon } &{}(b\le d, \delta \ge \lambda ^{b-d-1}), \\ {\delta ^{\frac{d}{b}} \lambda ^{ d -\frac{d}{b}+ \epsilon }} &{}(b\ge d+1, \delta \ge \lambda ^{d+1-b}). \end{array}\right. } \end{aligned}$$

We use the first of these alternatives when $\delta \ge \lambda ^{1-d}$, and otherwise we use the second. In particular by writing $a= d+1-b$ if $\delta \ge \lambda ^{1-d}$ and $a = b-d-1$ otherwise, we obtain (6.2). $\square$

## Appendix A. Borel-Cantelli lemma

At several points we use versions of the following argument to turn a bound on average over $(\beta _{ij})$ into a bound for almost all $\beta$.

Let $\beta =(\beta _{ij})$ be as in Definition 1.1. That is, either $\beta _{ij}=\delta _{ij}\beta _i$ with $\beta _i\in [1,2]$ for each *i*; or $\beta _{ij} = \delta _{ij} +h_{ij}$ for each $1\le i,j \le d$ and some $h_{ij}=h_{ji} \in \left[ -\frac{1}{10d^2} , \frac{1}{10d^2} \right]$.

We consider a function $\Phi ( \beta , \vec {P} )$ that depends on $(\beta _{ij})$ and also a list of parameters $P_1,\dotsc ,P_k$, for some $k\in {\mathbb {N}}$. In applications these parameters will be integer powers of 2, constrained by some inequalities. For example in the proof of Lemma 3.3 we should let $\vec {P}$ take values in the set

$$\begin{aligned} S=\left\{ (N,T, Q_1,\dotsc ,Q_d)\ \in (2^{{\mathbb {Z}}})^{d+2} : 2\le Q_j \le N, \tfrac{1}{4N}\le T \le N^\kappa \right\} , \end{aligned}$$

and put

$$\begin{aligned} \Phi ( \beta , \vec {P} )= \int _{1}^{2} \dots \int _{1}^{2} \int _0^T \Lambda _{Q_1}(t) \Lambda _{Q_2}(\beta _2 t) \dots \Lambda _{Q_k}(\beta _k t)\,dt \,d\beta _2 \dots d\beta _k. \end{aligned}$$

### Lemma A.1

Let $\Phi$ be as above. Assume that $\vec {P}$ takes values in a subset $S\subset (2^{{\mathbb {Z}}})^{k}$ such that for any $\epsilon >0$ we have

$$\begin{aligned} \sum _{\vec {P}\in S} P_1^{-\epsilon } <\infty . \end{aligned}$$

If $\Psi$ is some real function of $\vec {P}$ with

$$\begin{aligned} \int |\Phi ( \beta , \vec {P} )| \, d\beta \le \Psi (\vec {P}), \end{aligned}$$

then for every $\vec {P}\in S$, for generic $\beta$ we have

$$\begin{aligned} \Phi ( \beta , \vec {P} )\lesssim _{\beta ,\epsilon } P_1^{\epsilon } \Psi (\vec {P}). \end{aligned}$$

The lemma follows at once from the standard statement of the Borel-Cantelli lemma.

## Footnotes

1 If $\chi$ and $\psi$ are two cutoff functions, then $\Vert P_{\lambda ,\delta }^\chi \Vert _{L^2 \rightarrow L^p} \lesssim \Vert P_{\lambda ,\delta }^\psi \Vert _{L^2 \rightarrow L^p} + \Vert P_{\lambda -\delta ,\delta }^\psi \Vert _{L^2 \rightarrow L^p} + \Vert P_{\lambda + \delta ,\delta }^\psi \Vert _{L^2 \rightarrow L^p}$.

2 Here, we are using that, if $m_1(\Delta )$ and $m_2(\Delta )$ are two Fourier multipliers such that $|m_1| \ge |m_2|$, then $\Vert m_1(\Delta ) \Vert _{L^2 \rightarrow L^p} \ge \Vert m_2(\Delta ) \Vert _{L^2 \rightarrow L^p}$. This follows from Parseval’s theorem.
